# The rotavirus VP5*/VP8* conformational transition permeabilizes membranes to Ca^2+^

**DOI:** 10.1371/journal.ppat.1011750

**Published:** 2024-04-04

**Authors:** Marilina de Sautu, Tobias Herrmann, Gustavo Scanavachi, Simon Jenni, Stephen C. Harrison

**Affiliations:** 1 Department of Biological Chemistry and Molecular Pharmacology, Harvard Medical School, Boston, Massachusetts, United States of America; 2 Laboratory of Molecular Medicine, Boston Children’s Hospital, Boston, Massachusetts, United States of America; 3 Department of Cell Biology, Harvard Medical School, Boston, Massachusetts, United States of America; 4 Program in Cellular and Molecular Medicine, Boston Children’s Hospital, Boston, Massachusetts, United States of America; 5 Howard Hughes Medical Institute, Harvard Medical School, Boston, Massachusetts, United States of America; University of California at Los Angeles, UNITED STATES

## Abstract

Rotaviruses infect cells by delivering into the cytosol a transcriptionally active inner capsid particle (a "double-layer particle": DLP). Delivery is the function of a third, outer layer, which drives uptake from the cell surface into small vesicles from which the DLPs escape. In published work, we followed stages of rhesus rotavirus (RRV) entry by live-cell imaging and correlated them with structures from cryogenic electron microscopy and tomography (cryo-EM and cryo-ET). The virus appears to wrap itself in membrane, leading to complete engulfment and loss of Ca^2+^ from the vesicle produced by the wrapping. One of the outer-layer proteins, VP7, is a Ca^2+^-stabilized trimer; loss of Ca^2+^ releases both VP7 and the other outer-layer protein, VP4, from the particle. VP4, activated by cleavage into VP8* and VP5*, is a trimer that undergoes a large-scale conformational rearrangement, reminiscent of the transition that viral fusion proteins undergo to penetrate a membrane. The rearrangement of VP5* thrusts a 250-residue, C-terminal segment of each of the three subunits outward, while allowing the protein to remain attached to the virus particle and to the cell being infected. We proposed that this segment inserts into the membrane of the target cell, enabling Ca^2+^ to cross. In the work reported here, we show the validity of key aspects of this proposed sequence. By cryo-EM studies of liposome-attached virions ("triple-layer particles": TLPs) and single-particle fluorescence imaging of liposome-attached TLPs, we confirm insertion of the VP4 C-terminal segment into the membrane and ensuing generation of a Ca^2+^ "leak". The results allow us to formulate a molecular description of early events in entry. We also discuss our observations in the context of other work on double-strand RNA virus entry.

## Author summary

To infect a cell, non-enveloped viruses must have a mechanism to transfer their genomes across a membrane. The double-strand RNA (dsRNA) viruses deliver into the cytosol not just their genomes but an intact subviral particle (diameter ~70 nm). Rhesus rotavirus (RRV) particles attach through a spike-like outer-layer protein, VP4 (cleaved to VP5* and VP8*), then wrap themselves in membrane at the cell surface, producing a small, cytosolic vesicle surrounding each virion, from which the subviral particles escape directly. Loss of Ca^2+^ from the entering virion always precedes escape. In previous work we found that a conformational change in VP5*/VP8* projects the C-terminal "foot" domain of VP5* outward. We now show, by combining cryo-EM and single-particle optical microscopy of RRV particles interacting with liposomes, that the outward-projected foot penetrates the lipid bilayer, permeabilizing it to Ca^2+^. The new experiments thus recapitulate the initial step in particle escape and show that the conformational change must occur while the spike protein is membrane-attached, to couple the VP5* conformational change with membrane insertion. These findings bring us closer to a full molecular description how a large, subviral particle (about 70 nm diameter) can be delivered into a target host cell.

## Introduction

Entry of non-enveloped viruses into animal cells requires transfer across a cell membrane, either at the cell surface or from an internal compartment. An important distinction is between those viruses for which only the genome crosses the membrane and those for which a large, subviral particle enters the cytosol, with varying degrees of uncoating thereafter. The former class includes many small, positive-strand RNA viruses, such as caliciviruses [1] and picornaviruses [2], which appear to generate a transfer channel for their single-strand RNA genome from multiple copies of a small, internal protein. The latter class includes adenoviruses, which penetrate through an endosomal membrane and ultimately release their double-strand DNA genome at a nuclear pore [3,4], the polyomaviruses, which penetrate from the endoplasmic reticulum (ER) [5–7], and the double-strand RNA (dsRNA) viruses.

**Fig 1:**
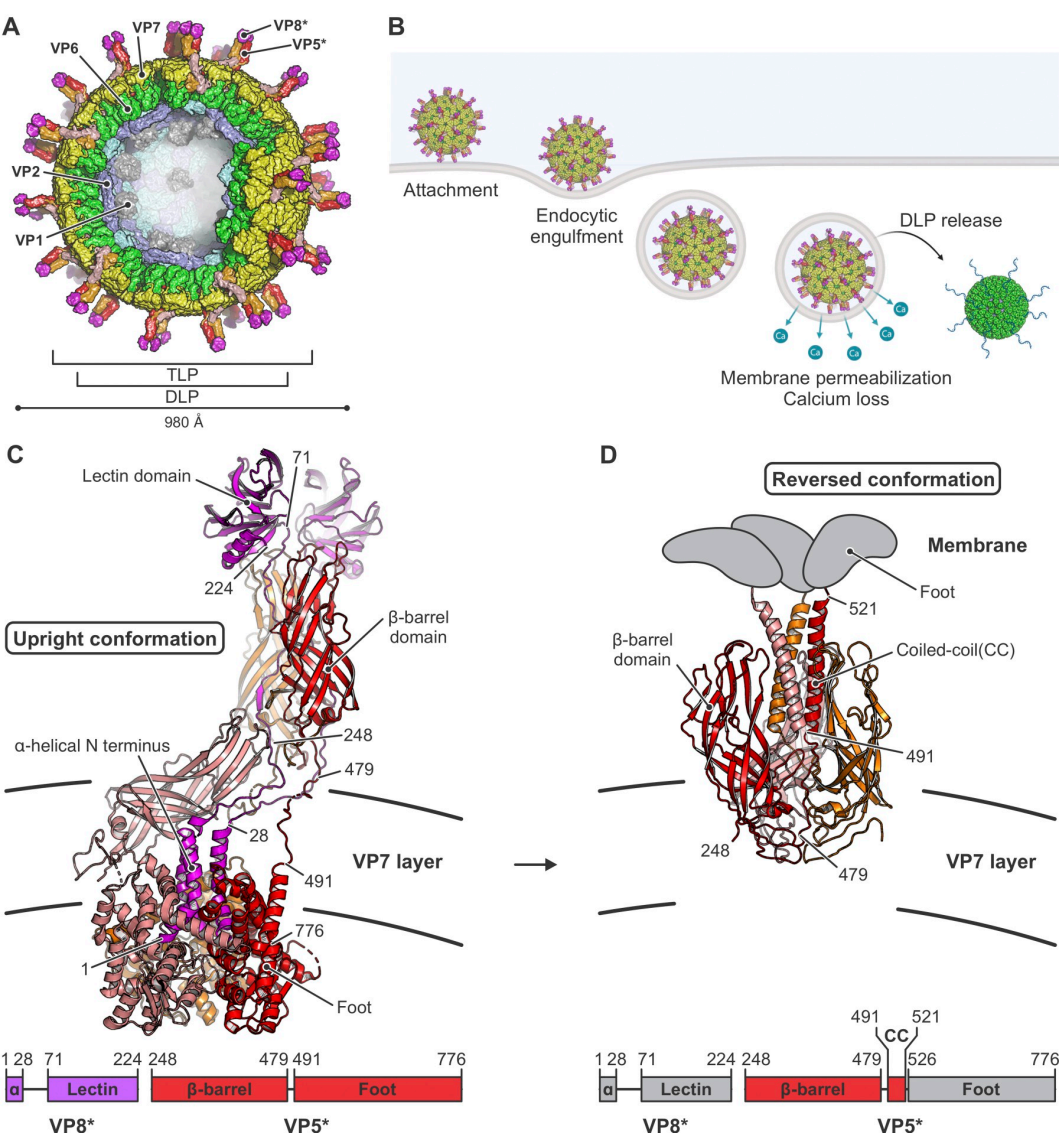
Rotavirus structure, entry, and conformational changes of its membrane-penetration protein VP4 (VP5*/VP8*). (A) Schematic illustration of an infectious rotavirus particle. Subunits are shown in surface representation and the particle is partially cut to allow inside view. The “double-layer” particle (DLP) is composed of VP2 (VP2A and VP2B, colored in blue and cyan, respectively) and VP6 (green). The outer layer of the “triple-layer” particle (TLP) also contains VP7 (yellow) and VP4 (VP5*/VP8* after proteolytic cleavage). VP5* is in red, orange and salmon; VP8*, in magenta; VP1, the capsid-bound RNA-dependent RNA polymerases (RdRp), in gray. The RNA cap-generating VP3, which has not been localized at a defined position in the virion, is not shown. (B) Schematic drawing of steps in rotavirus entry, summarizing observations from live-cell imaging [24,26,61] and biochemical and structural studies [16,18]. Entry requires trypsin-catalyzed cleavage of the VP4 spike protein into VP5* and VP8*. VP8* binding to glycolipid headgroups allows the TLP to attach to the surface of a host cell. This interaction, potentially in concert with membrane engagement of hydrophobic loops on VP5* (see panels C and D), allows the particle to enclose itself in an inward-budding vesicle. Subsequent steps include loss of Ca^2+^ from the vesicle interior, dissociation of VP7 and VP8*/VP5* from the DLP, free diffusion of the DLP in the cytosol, and initiation of RNA transcription. (C) Structure of the VP5*/VP8* spike in upright conformation (PDB-ID 6WXE) [18], colored as in (A). The linear domain organization of the upright conformation is shown at the bottom. (D) Structure of the VP5* spike in reversed conformation (PDB-ID 6WXG) [18], colored as in (A). The linear domain organization of the reversed conformation is shown at the bottom. Residues of the foot VP5* domains that were extruded from the VP7 layer cavity upon structural transition are shown schematically in gray.

Rotaviruses, like most other double-strand RNA (dsRNA) viruses, initiate infection of a cell by delivering an intact, inner capsid particle into the cytosol [8]. The two protein shells of this "double-layer particle" (DLP) enclose the 11 distinct segments of the dsRNA genome [9], each associated with an RNA-dependent RNA polymerase (RdRp) [10,11] and an RNA cap-generating activity [12,13]. On an infectious virion, a "triple-layer particle" (TLP), an outer protein shell surrounds the DLP (Fig 1A). Its role is to effect delivery of the DLP, which once liberated into the cytosol begins promptly to transcribe the dsRNA genome segments and to extrude them as capped mRNAs (Fig 1B).

Current evidence suggests that the two protein components of the outer layer, VP4 and VP7, have distinct functions in DLP delivery [14–18]. VP4, activated by proteolytic cleavage into VP8* and VP5*, is the principal membrane-interacting partner; VP7, held together by Ca^2+^ ions at its trimer interfaces, is a Ca^2+^-sensitive “clamp” that anchors VP4 onto the underlying DLP. For mammalian rotaviruses, cell attachment generally requires interaction of the VP8* moiety of VP4 with a glycolipid headgroup [19–23]. For rhesus rotavirus (RRV) entering BSC-1 or SVG-A cells, uptake of the virus particle into a small vesicle does not require clathrin, dynamin, or related activities; the virus particle appears to produce its own engulfment by wrapping the plasma membrane around itself [24]. Clathrin-mediated endocytosis is probably an entry route for other strains and in other cell types (reviewed in [25]), but prompt clathrin uncoating would leave the virion in a small, closed vesicle, just as in the wrapping just described. Accompanying or following the engulfment step is a large-scale conformational rearrangement of the projecting VP4 trimer (cleaved to VP8* and VP5*) from an asymmetric, “upright” conformation (Fig 1C) to a threefold symmetric, “reversed” conformation (Fig 1D) [18]. The transition takes place while the protein remains anchored by VP7 on the surface of the virus particle. A major consequence of the reversal is projection outward of the ~250 amino-acid residue “foot” segment of the VP5* polypeptide chain (Fig 1D). From our published structures and low-resolution cryogenic electron tomography (cryo-ET) [18], we concluded that the three copies of this foot segment probably insert into the surrounding, target-cell derived membrane, where they permeabilize the membrane to Ca^2+^ ions. Loss of the Ca^2+^ ions that stabilize VP7 trimers precedes delivery of DLPs into the cytosol [26]; subsequent steps are then dissociation of the outer layer, and escape of the DLP into the cytosol (Fig 1B).

We report here that we can recapitulate with liposomes early steps in the entry process–attachment to glycolipid headgroups and insertion of the projected foot domain into the lipid bilayer, as visualized by cryogenic electron microscopy (cryo-EM). We show by single-particle fluorescence imaging that this same interaction leads to passage of Ca^2+^ across the liposome membrane, but not to further disruption of the liposome. We relate these observations to the entry steps observed by live-cell imaging.

## Results

### TLP permeabilization of liposomes to Ca^2+^ ions

**Fig 2:**
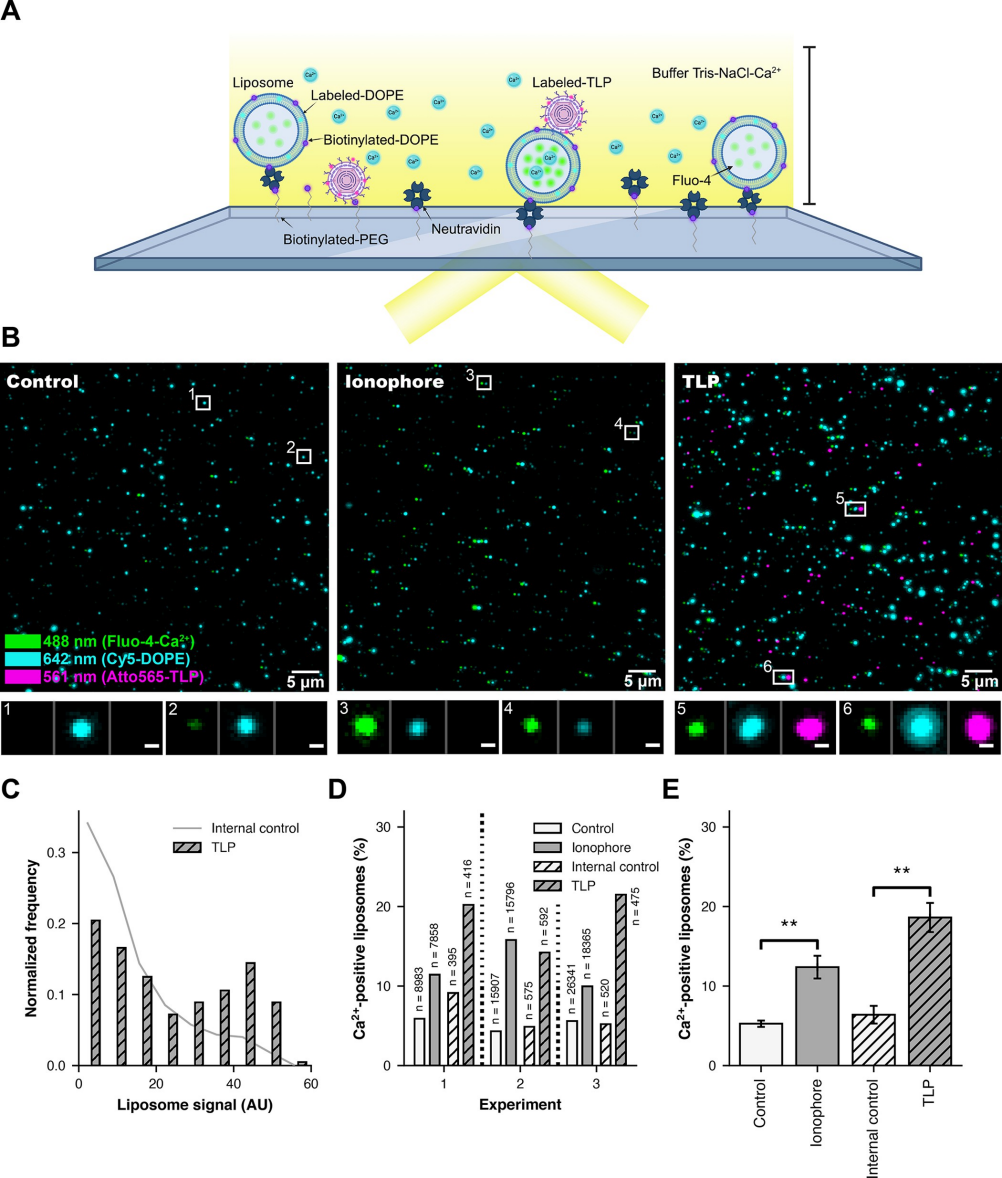
Interaction with rotaviruses makes liposomes Ca^2+^ permeable. (A) Schematic overview of the TIRF experimental setup. (B) Representative fluorescence images, showing a field of view for the control (left, liposomes only), ionophore (center, liposomes incubated with ionomycin) and TLP (right, liposomes incubated with TLPs) samples. The 640 nm channel (Cy5-DOPE, liposome signal) is in cyan and shifted 7 pixels to the right in all images to visualize colocalization; the 488 nm channel (Fluo-4, Ca^2+^ sensor signal) is in green; the 561 nm channel (Atto 565 NHS ester dye, virus signal) is in magenta and shifted 14 pixels to the right in the TLP sample. The scale bar corresponds to 5 μm. Representative examples of liposomes are boxed (1–6) and displayed beneath the images at higher magnification (scale bar = 0.5 μm) with the three channels shown next to each other. (C) Normalized frequency of the liposome intensity signal in arbitrary units (AU) from the TLP sample (experiment 1). The grey line shows the distribution for internal control liposomes in the TLP sample (liposomes that did not colocalize with a virus particle in the TLP sample) (n = 28,985). The hatched grey bars show the distribution for the liposomes that colocalized with a virus (n = 415). (D) Ca^2+^-positive liposomes (%) for the control sample (white bars), the ionophore sample (grey bars), the internal control in the TLP sample (liposomes in the TLP incubated sample that do not colocalized with TLP, hatched white bars), and TLP sample (liposomes that colocalized with TLP, hatch grey bars) from experiments 1, 2 and 3, respectively. "Ca^2+^-positive liposomes" means percent of the liposomes that had Ca^2+^ signal intensity greater than 1.5 standard deviations from the mean in the corresponding internal control sample (see red dashed lines in S2A and S2C Fig for the cutoffs). n = total number of liposomes in each condition. (E) Average percent (%) Ca^2+^-positive liposomes for the control sample (white bar), the ionophore sample (grey bar), the internal control in the TLP sample (hatched white bar), and TLP sample (hatched grey bar) from the three independent experiments (S1 and S2 Figs). Error bars are the standard error calculated from the mean of the independent experiments. Statistical analysis used a t-test between samples. * = p<0.05; ** = p<0.01; *** = p<0.001.

Loss of Ca^2+^ from TLPs is an early step following engulfment at the cell surface [26]. We tested whether an interaction of virions with a membrane could lead to Ca^2+^ permeability, without contribution of potential cellular factors, using liposomes containing, in their lumen, a soluble, Ca^2+^-sensitive fluorophore (Fluo-4) and, in their bilayer, a fluorescently labelled lipid (Cy5-DOPE) for visualization, as well as biotinylated lipids for attachment to an avidin-coated coverslip. We prepared the liposomes in the absence of Ca^2+^ and used a lipid composition similar to that of mammalian-cell plasma membranes supplemented with 2.5% GD1a, a validated rotavirus attachment factor [19–23]. In the absence of Ca^2+^, we established the background fluorescence of the incorporated Fluo-4 dye. We incubated the liposomes with fluorescently labeled TLPs (Atto 565) in Ca^2+^-containing buffer, then deposited the sample onto a coated coverslip and recorded signal at appropriate wavelengths by total internal reflection fluorescence (TIRF) microscopy (Figs 2A, 2B, S1 and S2). Thus, the intensities of the diffraction-limited spots in each of the three recorded channels reported, respectively, the presence of liposomes, the presence of Ca^2+^-liganded Fluo-4 (and hence permeabilization of the liposome to Ca^2+^), and the attachment of TLPs. In a parallel control experiment, we used ionomycin, a Ca^2+^ ionophore, to permeabilize similarly prepared liposomes.

The distribution of intensities for the lipid marker (Cy5-DOPE) showed that the liposomes varied in size over an extended range but that this range was very similar for all samples and experiments (S1B Fig). The larger liposomes were more likely to have a colocalized TLP, suggesting that the particles had associated preferentially with the larger liposomes, as might be expected from their greater cross section (Fig 2C). Some control liposomes, incubated with neither ionomycin nor TLP, showed Ca^2+^ positive signal, corresponding to random "leaks", which varied somewhat in frequency from sample to sample (Figs 2B, 2D and S2). Addition and binding of TLPs yielded a markedly enhanced frequency of Ca^2+^ penetration (Fig 2D), at levels comparable to the enhancement by ionomycin (Figs 2D, 2E and S2). For the overall effect shown in Fig 2D, we calculated the mean number of Ca^2+^-positive liposomes by counting the number of particles that showed a Ca^2+^ signal with an intensity of 1.5 standard deviations above the mean intensity in the corresponding internal control calculated from the TLP-free liposomes in the same field as the TLP-associated liposomes (S1 and S2 Figs). For the data summary in Fig 2E, we averaged the results of the three independent experiments.

**Fig 3:**
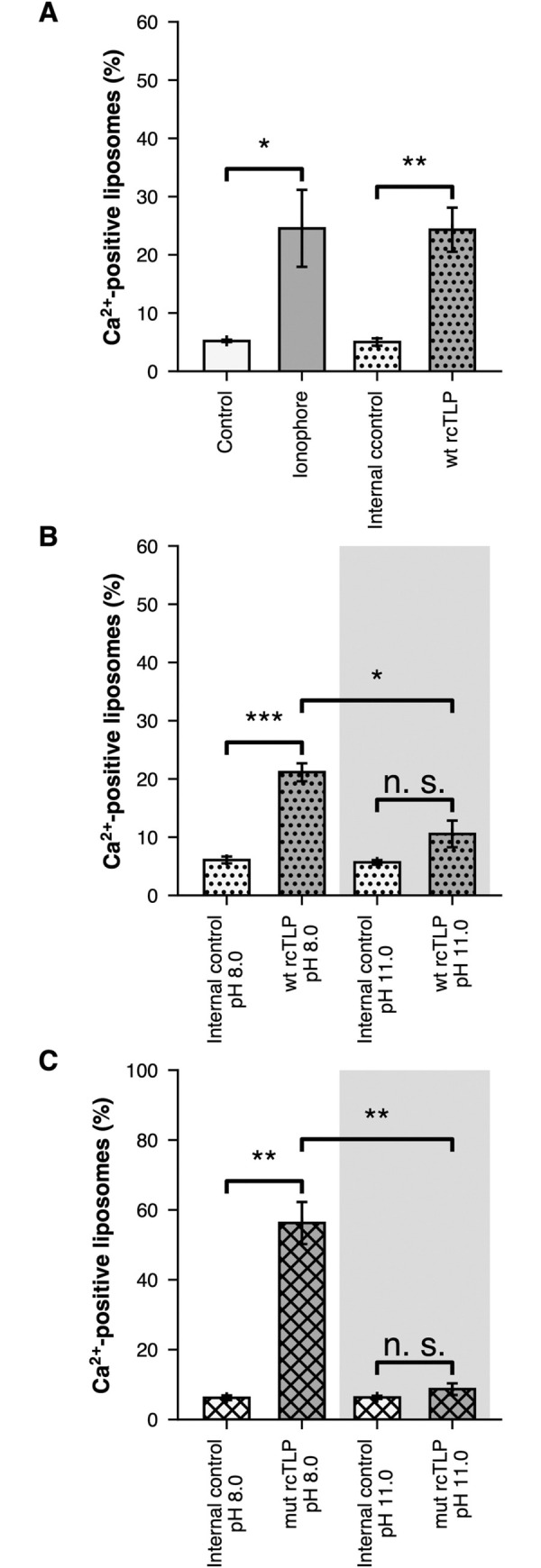
Exposure of rotaviruses to pH 11.0 before liposome incubation prevents Ca^2+^ permeabilization. (A) Average percent (%) Ca^2+^-positive liposomes, from at least three independent experiments (S3 and S7A Figs), for control liposomes (white bars), ionophore-exposed liposomes (grey bars), internal control in the wt rcTLP sample (liposomes in the wt rcTLP sample that did not colocalize with wt rcTLP: dotted white bars) and wt rcTLP sample (liposomes that colocalized with wt rcTLP: dotted grey bars). (B) Average % Ca^2+^-positive liposomes, from three independent experiments (S5 and S7B Figs), for the internal control and wt rcTLP sample pre-incubated at pH 8.0 (dotted white and dotted grey bars, respectively), and for the internal control and the wt rcTLP sample and wt rcTLP pre-incubated at pH 11.0 (dotted white and dotted grey bars on grey background, respectively). (C) Average % Ca^2+^-positive liposomes, from three independent experiments (S6 and S7C Figs), for the internal control and the mutant rcTLP sample pre-incubated at pH 8.0 (crossed hatched white and crossed hatched grey bars, respectively), and for the internal control and the mutant rcTLP sample pre-incubated at pH 11.0 samples (crossed hatched white and crossed hatched grey bars on grey background, respectively). "Ca^2+^-positive liposomes" designates liposomes that had a Ca^2+^ signal intensity greater than 1.5 standard deviations from the mean in the corresponding control sample (see red dashed lines in S3A, S3C, S5A, S5C, S6A and S6C Figs for the cutoffs). Error bars are the standard error calculated from the mean of the independent experiments. Statistical analysis used a t-test between samples. n.s. = p>0.05; * = p<0.05; ** = p<0.01; *** = p<0.001.

We also used the method just described to examine Ca^2+^ permeabilization by recoated particles (rcTLPs)—DLPs recoated *in vitro* with purified VP7 and VP4. These particles have physical properties and specific infectivities comparable to those of authentic TLPs as purified from infected cells [15,26,27]. As shown by the experiments summarized in Figs 3A and S3, Ca^2+^ permeability induced by rcTLPs is, like other most other properties of these particles, essentially the same as permeability induced by authentic TLPs (Fig 2E).

### Ca^2+^ permeabilization requires foot insertion

We tested whether insertion of the VP4 foot into the lipid bilayer is essential for transit of Ca^2+^ into the liposome by eliciting spike reversal with a short exposure to high pH and by comparing results obtained with particles recoated with wild-type (wt) VP4 and with a mutant, "foot-locked VP4", in which a disulfide bond prevents unfolding and outward projection of the foot [18]. Brief incubation of RRV particles at pH 11 followed by return to pH 8 induces complete reversal of wild-type (wt) VP4 on both authentic and recoated TLPs [18,28]. The foot-locked mutant (mut) VP4 transitions to an intermediate structure, in which the projecting domains rearrange, but the foot remains in place [18]. Recoating with this mutant VP4 is even more efficient than with the wild-type protein, perhaps because of stabilization of the foot as the disulfide forms during the recoating step (S4 Fig), but owing to incomplete disulfide formation, the mutant-recoated particles retain some residual infectivity (S4 Fig). Classification of cryo-EM subparticles shows that about one-third of the VP4 spikes do reverse at pH 11 and that the efficient recoating has resulted in essentially complete occupancy (no "empty-position" class), rather than the 40–50% level for wild-type VP4 [18].

Fig 3B and 3C show experiments that compare Ca^2+^ permeabilization by wild-type (wt rcTLP) and disulfide-mutant (mut rcTLP) particles. The substantially more efficient recoating with mutant VP4 results in an even higher proportion of permeabilized rcTLP-associated liposomes than for wild-type VP4. Therefore, to inactivate all reversible VP4 spikes (i.e., those in which the disulfides had failed to form), we pulsed the particles at pH 11 for 30 mins and returned them to pH 8 for the liposome incubation. Fig 3B shows that this treatment inactivated wild-type VP5*/VP8* spikes, and Fig 3C shows that it also eliminated permeabilization by the mutant-recoated particles. Thus, neither the intermediate VP5*, with hydrophobic loops on the β-barrel domain well exposed but the foot still anchored in the particle, nor the fully reversed VP5* has the capacity to allow Ca^2+^ to cross the bilayer. That is, for Ca^2+^ permeabilization, the VP5*/VP8* conformational change needs to occur while the particle is bound to the membrane, probably (as indicated by the cryo-EM analysis that follows) so that coiled-coil formation can provide some of the energy needed to drive part or all of the foot into (and potentially through) the lipid bilayer.

Despite permeabilization to Ca^2+^, the liposomes remained impermeable to Fluo-4. Thus, the interaction with rotavirus particles did not, under these conditions, disrupt liposome integrity. We can conclude that binding of an RRV TLP to a membrane containing an appropriate sialic-acid receptor permeabilizes the liposome membrane to Ca^2+^ ions, without otherwise rupturing the lipid bilayer. If passage of Ca^2+^ is through a pore-like structure, then retention of Fluo-4 sets an upper limit of ~15 Å for the pore diameter.

### Cryo-EM analysis of TLPs with liposomes

**Fig 4:**
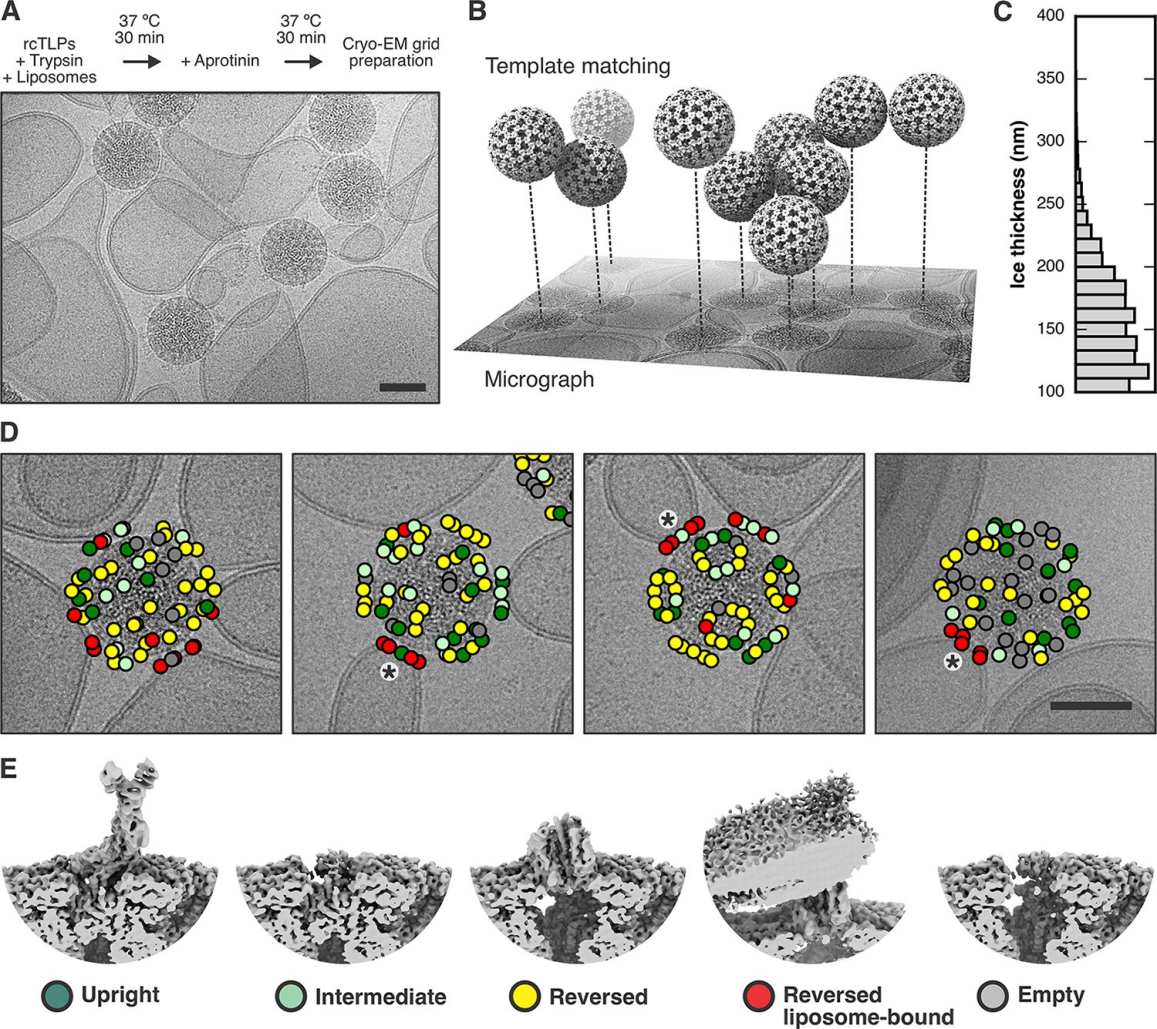
Cryo-EM analysis of rotavirus in the presence of liposomes. (A) Sample preparation scheme and representative micrograph showing rcTLP particles bound to liposomes. Scale bar: 50 nm. (B) Illustration of how template matching was used to locate virions in the micrographs and estimate their *z* position based on per-particle defocus fitting. (C) Histogram of estimated ice thickness of the cryo-EM samples. For each micrograph that contained at least two particles, we calculated the largest *z* difference and added 100 nm (approximately two times the particle radius). (D) Mapping of spike positions and their class assignment onto the micrographs of liposome-bound viruses, color-coded as shown in panel (E). The asterisks indicate a cluster of VP5* trimers around the five-fold axis of the virus. Scale bar: 50 nm. (E) Representative reconstructions of the different spike conformations after subparticle classification.

We examined whether the upright-to-reversed conformational change indeed drives the foot into the membrane, as proposed in our earlier paper [18] and inferred in the previous section, by visualizing with cryo-EM the interaction of TLPs with lipid-bilayer membranes (Fig 4A). We prepared liposomes as in the experiments above, but without Fluo-4 and without the membrane label. We used recoated particles (wt rcTLPs), because of previous observations that VP4 on rcTLPs appeared to undergo more readily a spontaneous transition from upright to reversed [18]. We recorded four cryo-EM data sets on a Thermo Fisher Titan Krios operated at 300 kV and equipped with a Gatan Bioquantum energy filter and K3 detector (S1 Table).

We found the position of viral particles on our micrographs by template matching from cisTEM [29,30]. By refining the local defocus for each particle, we obtained its relative *z* position within the vitreous ice (Fig 4B). Fig 4C shows a histogram of the largest *z* difference plus 100 nm (approximately two times the radius of a virion), for micrographs from which we picked at least two virus particles. We did not directly measure the ice thickness [31], but we estimated from the observed distribution, that the ice thickness of our samples varied between 100 and 250 nm.

Images in Fig 4D show that many particles appeared to have extended contacts with liposomes, but many of these were with liposomes of radius ≥300 nm and hence substantially flattened in the ice. As a consequence of flattening [32,33], the bilayer would have been forced to curve away from the particle, not just in the plane of the ice but in the directions normal to it, probably peeling the membrane away from the particle during blotting (S8 Fig). Moreover, as the suspension thinned, attached particles would have migrated to the periphery of the flattening liposome in order to remain in solution, distorting the liposome in the process. Thus, the invaginations seen at the more extended contacts appear to be largely within the plane of the image and of limited extent above and below it, and our images probably do not represent the full extent of contact achieved in the preparation. We could detect no evidence of liposome disruption when TLPs bind or when the interacting VP5* has transitioned from upright to reversed conformation.

An icosahedral reconstruction carried out after merging virus images from all four datasets resulted in a density map with an overall resolution of the viral shell of 2.43 Å (S9 Fig and S1 Table). As described in Methods and in S10 and S11 Figs, we then used subparticle classification (S10D and S10E Fig), starting with a total of 4,550,760 masked VP4 subparticles, to separate upright, intermediate, reversed, and empty VP5*/VP8* spikes positions (classifications #1 and #2, S12 Fig and S2 Table) and to distinguish membrane-engaged from non-engaged spikes (classifications #3 and #4, S13 Fig and S3 Table). Two relatively heterogeneous classes showed membrane-engaged reversed spikes, with apparently variable angles of spike contact (S13A Fig, classification #3). A further classification step then yielded two maps from 56,593 and 70,018 subparticles (classes 5 and 6), respectively, with relatively well-defined lipid-bilayer density (S13B Fig, classification #4). Virions that contributed subparticles to these two classes had on average a higher percentage of occupied spike positions (liposome-unbound rcTLPs = 48.9% empty VP5*/VP8* positions, liposome-bound rcTLPs = 36.9% empty VP5*/VP8* positions) (S4 Table), showing that those particles had bound preferentially to liposomes. Even within these classes, variability of curvature at positions at which the bilayer bent away from the particle blurred the membrane profile around the circumference of the contact. Local resolution estimates for classes 5 and 6 showed a well-resolved, virion-bound VP5* β-barrel trimer with its central coiled-coil domain and a poorly resolved lipid bilayer (S14 Fig).

**Fig 5:**
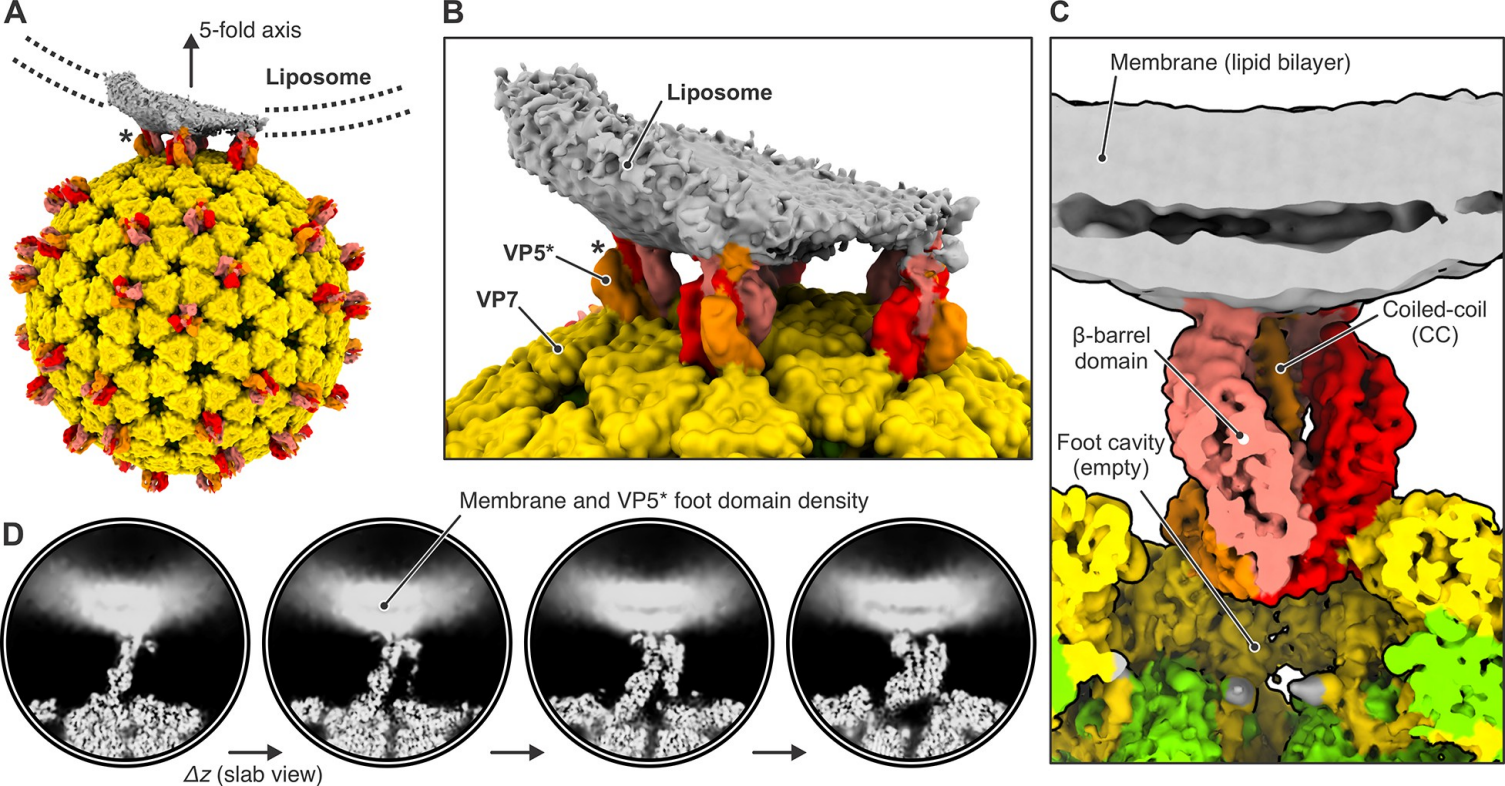
Cryo-EM reconstruction of membrane-bound VP5* in the reversed conformation. (A) Reconstruction of class 6 (S13B Fig) in context of the full virus. The map was low-pass filtered at 8.0 Å resolution. The outer-shell VP7 is colored yellow; VP5* is colored red, orange, and salmon; membrane density is gray. The asterisk denotes the VP5* trimer on which the subparticle classification was focused. The direction of one of the icosahedral five-fold axis is indicated. (B) View around the five-fold axis showing multiple VP5* trimers interacting with liposome. The asterisk denotes the VP5* trimer on which the subparticle classification was focused. (C) Close-up view of the membrane-bound VP5* trimer. The map is partially cut. (D) Slab views of the reconstruction showing diffuse lipid bilayer and membrane-embedded VP5* foot domain density.

The class 6 reconstruction has pronounced lipid bilayer density on one side of the reversed VP5* and somewhat fuzzier density on the side opposite (Fig 5). The angle between the curving bilayer and the VP5* trimer axis shows that this class represents a reversed spike near the edge of the contact. The side with a well-defined profile faces the nearest fivefold axis (Fig 5A and 5B). A fivefold axis may be close to the center of many of these membrane contacts, because the five surrounding spikes can offer a compromise between a minimally distorted membrane region and a set of membrane contacts stable enough to resist the forces during thinning of the aqueous film, as described above (see diagram in S8 Fig). We verified that many of the subparticles contributing to this class indeed came from reversed VP5* spikes related by a five-fold axes, by mapping the class assignment onto the original micrographs (Fig 4D and 4E). The coiled-coil at the C-terminal end of the VP5* β-barrel domains extends just to the inner lipid headgroup layer, and diffuse density extends beyond it into the bilayer profile, but peeling away of the bilayer leaves nearly all the engaged spikes at the periphery of the contact, and the resolution leaves uncertain the molecular features of the likely membrane-penetrating elements (Fig 5C and 5D).

**Fig 6:**
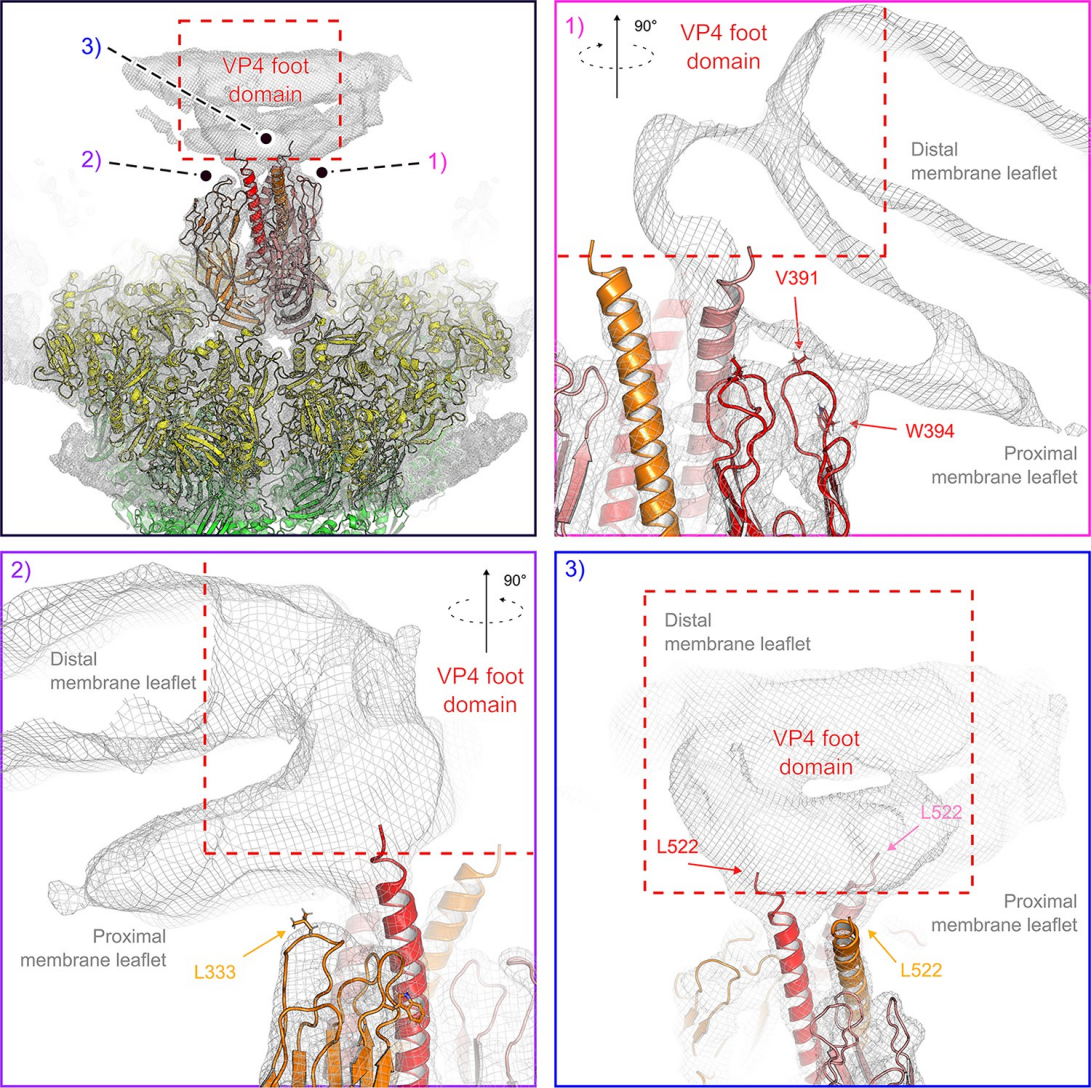
Interactions between the reversed VP5* trimer and the membrane. (A) Placement of the rotavirus-bound VP5* trimer structure in reversed conformation (PDB-ID 6WXG) [18] into the density map of the class 6 reconstruction (Fig 5). Density is shown as gray mesh, docked viral protein subunits are shown in ribbon representation and colored yellow (VP7), red, orange and salmon (VP5*). Close-up views are shown for the hydrophobic loops at the tip of one VP5* β-barrel that interact with the proximal membrane leaflet (1), for the hydrophobic loops at the tip of a second VP5* β-barrel that interact with the proximal membrane leaflet (2), and for the trimeric VP5* coiled-coil extending into the membrane (3).

The reversed-spike structure (PDB-ID 6WXG) [18] can be docked with confidence into the observed subparticle density (Fig 6). The tips of the VP5* β barrel are hydrophobic loops [15]. Two of the three tips contact the liposome bilayer, while its curvature from flattening has probably eliminated a contact from the third. Mutations that reduce the hydrophobicity of these loops inhibit viral entry [15], and interpretation of tomographic reconstructions of TLPs entering at the thin edges of BSC-1 cells has suggested that these loops interact with membrane directly [18]. Our current reconstruction is consistent with this interpretation and suggests that the interaction might already be present in the proposed intermediate state, seen with the foot-locked mutant, in which the β-barrel domains have shifted into the threefold arrangement found in the reversed structure, but the foot domains have not yet begun to project outward from their positions in the pre-attachment TLP [18].

## Discussion

Our previous study combined cryo-EM single-particle analysis with cryo-ET of engulfed virions entering cells to show that VP5* on the surface of an attached TLP can undergo a transition from an upright to a reversed conformation without dissociating from the particle, causing its C-terminal foot domain to project toward the apposed membrane (Fig 1B and 1C) [18]. We have now shown, by analysis of individual spike positions on cryo-EM images of virions attached to liposomes, that VP5* trimers, in reversed conformation, interact with the lipid bilayer, recapitulating the interaction inferred from icosahedral averaging of much lower resolution cryo-ET images (Figs 4 and 5). The reconstruction shows that the central coiled-coil domain protrudes into the lipid bilayer, although flattening and distortion of the liposomes have prevented us from resolving protein structure within the bilayer. Under essentially identical conditions, TLPs that attach to similar liposomes allow Ca^2+^ ions to pass into the liposomes without destroying their integrity. We can equate this process with the early loss-of-Ca^2+^ step detected by live-cell imaging [24]. In the experiments here, Ca^2+^ enters a liposome; during viral entry, it leaves the vesicle surrounding the TLP, driven by the concentration gradient from vesicle to cytosol. Insertion of the foot domain into the bilayer requires that the particle be membrane-bound when the conformational change from upright to reversed takes place.

Combining these results with those from earlier work, we summarize as follows our current understanding of the molecular events accompanying RRV entry.

1. Initial attachment to sialoglycolipids on the surface of a cell is through VP8* at the apex of the VP8*/VP5* spike in its upright conformation [24,34].
2. Engulfment (disappearance from the cell surface into the cytoplasm) is complete within a few minutes for most of the attached particles. DLPs recoated with VP4 mutated in the hydrophobic loops of VP5* move from the cell surface into the cell interior, but fail to release into the cytosol [24] and fail to infect [15]. That is, engagement of the hydrophobic loops with the membrane bilayer appears not to be necessary for particle uptake but essential for later steps. Complete enclosure from an essentially flat lipid bilayer would require that interaction of the virion with the membrane liberate a total free energy of about 200 kcal/mol of vesicle (but substantially more for 30–40 nm liposomes) [35]. The K_d_ of RRV VP8* for sialic acid is 1.2 mM; a rough calculation of the free energy derived from binding, taking as a local concentration of sialic acid headgroups as the number of gangliosides within 50 Å of a VP8* site and the ganglioside concentration in the membrane as 1% of the phospholipids, suggests that the free energy of binding could indeed generate enough work (≥200 kcal/mol of particle) to drive complete wrapping of the membrane, assuming high VP4 occupancy. In practice, gangliosides and other sphingolipids tend to cluster transiently into so-called "lipid rafts", so that the local concentration might be considerably higher than the cell-surface average.
3. VP4 on the surface of a virus particle can transition spontaneously into its reversed conformation. Why do the engulfed hydrophobic-loop mutants fail to release and infect? A possible explanation is that when the hydrophobic loops cannot engage the membrane, the projected foot fails to insert, and the reversal is futile. Formation of the central coiled-coil presumably drives foot extrusion, but loop engagement (or some attachment stronger than VP8* binding to ganglioside headgroups) may be necessary to resist the kinetic barrier to membrane insertion. This explanation is consistent with the published *in vitro* observation that release of cleaved VP4 (i.e., VP8*/VP5*) from TLPs (by chelating Ca^2+^) in the presence of liposomes leads to liposome association of VP5* trimers, but not if liposomes are added later [36]. It also follows from our finding here, that to permeabilize the membrane to Ca^2+^, reversal and projection of the foot must occur while the particle is membrane bound; pre-reversed VP5* is not sufficient.
4. What initiates the reversal? The interface between the VP8* lectin domain and VP5* is modest, and that domain alone would probably dissociate readily from its VP5* partner. The VP8* N-terminal, coiled-coil anchor in the foot will prevent full dissociation from the virion. However, even if the lectin domain fluctuates on and off, the fluctuation will expose the VP5* hydrophobic loops, which can then engage a membrane to which VP8* has attached and prevent return of VP8* to its docking site. This picture is consistent with our suggestion, above, that hydrophobic loop engagement is essential for foot insertion. It is also consistent with our finding for a human rotavirus vaccine-strain candidate, that mutations during passage, including one at the VP8*/VP5* interface, stabilize the upright conformer [37].
5. Foot insertion allows Ca^2+^ to cross the vesicle membrane. If reversal and membrane insertion occur before complete engulfment, Ca^2+^ will pass into the cytosol from the extracellular medium. It is indeed possible that Ca^2+^ signaling by this mechanism ordinarily accompanies rotavirus entry. Foot insertion within a closed vesicle will quickly deplete the contents of Ca^2+^, entraining outer-layer dissociation and subsequent events, which must include further disruption or perforation of the vesicle membrane.
6. DLPs recoated with VP7 and the foot-locked VP4 mutant that cannot undergo the full reversal have substantially lower infectivity than do those recoated with VP7 and wild-type VP4 (S4 Fig and [18]). Yet without the pH 11 pulse, incomplete disulfide formation leaves enough reversal-competent spikes to promote efficient Ca^2+^ permeabilization in our *in vitro* assay. The most likely inference from these observations is that a greater number of "active" spikes are required for the vesicle membrane disruption than the number necessary to allow Ca^2+^ transfer.

Orbiviruses, as represented by bluetongue virus, appear to have a similar mechanism for membrane insertion and endosome penetration [38,39]. Bluetongue virus enters through low-pH compartments, and the signal for conformational change is proton binding, probably by a cluster of histidines, rather than Ca^2+^ loss. The bluetongue virus subunit VP5 (the functional analog of rotavirus VP5*, but with a quite different, trimeric structure) undergoes, when the pH drops to 6 or below, a stepwise "unfurling" of an antiparallel coiled-coil, thereby forming a ~190 Å long stalk, at least part of which is a six-helix bundle [39]. Residues in the three loops at the distal tip of this bundle appear to insert into liposomes when the latter are mixed with virus particles *in vitro*. The mechanism for release of the core particle (the DLP equivalent) from an endosome may involve a wrapping of endosomal membrane around the low-pH altered virion, as suggested by images of particles interacting with liposomes in a format similar to the one used here [39].

The outer-layer proteins of orthoreoviruses undergo a series of conformational changes initiated by proteolytic removal of σ3 and extension of the receptor-binding protein σ1 (reviewed in [40]). The altered particle can then initiate penetration by releasing a pore-forming, myristoylated peptide (μ1N), generated by autolytic cleavage of the outer-shell protein, μ1 [41,42]. The pores formed *in vitro* in red blood cells or liposomes are about 50 Å in diameter, but they could in principle be larger when μ1N release occurs within an endocytic vesicle. A molecular mechanism for the ultimate release of a core particle into the cytosol is still uncertain.

The rotavirus infectious cycle includes another membrane-crossing step, when a newly assembled DLP, together with VP4, acquires its VP7 complement in the endoplasmic reticulum (ER). VP7, a glycoprotein, folds in the ER; inhibition of its synthesis or of its proper folding leads to accumulation in the ER of enveloped DLPs [43–45]. Recent cryo-ET studies under conditions of VP7 inhibition have captured VP4-decorated DLPs budding into the ER [46], presumably mediated by the virally encoded ER receptor, NSP4 (formerly NS28) [47]. Whether the resulting enveloped particles are an on-pathway intermediate or a dead-end consequence of VP7 inhibition remains to be established. It is possible that VP7 trimers participate in productive budding, rather than displacing an already complete bilayer from around a budded particle. It likewise remains possible that VP7 participates directly in the membrane disruption required for complete release of the DLP during viral entry—the step for which we now seek a molecular picture.

## Methods

### Cells, plasmids and constructs

MA104 cells (ATCC, type: MA104 clone 1, ATCC CRL2378.1, cat. # ATCC CRL2378.1) were grown in M199 medium (Thermo Fisher Scientific) supplemented with 25 mM 4-(2-hydroxyethyl)-1-piperazineethanesulfonic acid (HEPES) and 10% Hi-FBS (Thermo Fisher Scientific). BSC-1 cells (ATTC) were grown in DMEM (Thermo Fisher Scientific) supplemented with 10% Hi-FBS (Thermo Fisher Scientific). For VP4 expression, we cloned its full-length genomic sequence from serotype P5B [3], NCBI:txid444185) into a pFastbac expression vector. For VP7 expression, we cloned its full-length genomic sequence (G3 serotype, NCBI:txid444185) into a pFastbac expression vector and transformed *E*. *coli* DH10α cells. For bacmid generation, VP4 and VP7 vectors were transformed into DH10-Bac cells (ThremoFisher Scientific) and plated onto LB-agar plates supplemented with 50 μg/ml kanamycin, 7 μg/ml gentamycin, 10 μg/ml tetracycline, 100 μg/ml blue-gal and 40 μg/ml β-D-1-thiogalactopyranoside (IPTG).

### Reagent preparation

*Purification of rotavirus DLPs and TLPs*–We grew MA104 cells to confluency in 850 cm^2^ roller bottles in M199 medium supplemented with 10% FBS and 25 mM HEPES. Cells were infected with rhesus rotavirus (RRV, strain G3P5B [3]) at a multiplicity of infection (MOI) of 0.1. After incubation at 37°C for 24 h, the medium containing cells and debris was collected and frozen at -80°C for storage. To purify DLPs, we uncoated TLPs by adding EDTA pH 8.0 to a final concentration of 5 mM. After thawing, we pelleted cells and debris by centrifugation in a Beckman Coulter rotor JS 4.2 at 2,900 g for 30 min at 4°C. Supernatant was then removed and the pellet resuspended in 1 ml ice cold TNE buffer (20 mM Tris, 100 mM NaCl, 1 mM EDTA, pH 8.0) for DLP purification. We concentrated viral particles from the supernatant by ultracentrifugation in a Beckman Coulter rotor Ti-45 rotor at 224,500 g. The supernatant was then discarded and the pellet resuspended in 1–3 ml ice cold TNC and combined with the cell-debris fraction from the first centrifugation. We transferred the resulting suspension to a 15 ml conical tube and added TNC to a final volume of 4 ml. We added 4 ml of 1,1,2-trichloro-1,2,2-trifluoroethane (Freon 113) and inverted the tube 5–10 times. The suspension was centrifuged for 2 min in a Beckman Coulter rotor SX4750 at 200 g at 4°C. We removed the upper aqueous phase, transferred it to a new clean 15 ml conical tube and repeated the Freon extraction two more times. The resulting virus solution was layered on top of a 34.7–60.0% (w/v) CsCl gradient (1.26–1.45 g/ml). CsCl solutions were prepared in either TNE (DLP purification) or TNC (TLP purification). Gradients were spun at 4°C in a Beckman Coulter rotor SW 60 at 406,000 g for 2.5 h. Bands for TLPs and DLPs were harvested by side puncture. TLPs and DLPs were dialyzed against 2 L of TNC or TNE overnight. Viral particles were concentrated by pelleting at 4°C in a Beckman Coulter rotor SW 60 at 406,000 g for 1 h. Supernatants were removed with a 1 ml pipette and pellets resuspended in 100–200 μl of remaining buffer. Concentrations of DLPs were determined with a nanodrop measuring the absorption at 260 nm (extinction coefficient = 4.7028 g^-1^cm^-1^). TLP concentrations were measured by densitometry of VP6 bands on Coomassie stained SDS-PAGE gels against DLP standards ranging from 0.1 to 1.0 mg/ml.

*Purification of recombinant VP7* –We initially obtained baculovirus carrying the genetic information of full-length rhesus rotavirus VP7 (G3 serotype) from bacmid transfected Sf9 cells (Thermo Fisher Scientific). Sf9 cells were then inoculated with baculovirus and passaged in the same cells three times, with an incubation time of 72 h for each passage. Eight flasks of 500 ml Sf9 cells with approximately 2 million cells per ml were then infected with 13 ml of passaged virus stock solution and incubated for 72 h. Cells were harvested at 4°C by centrifugation in Beckman Coulter rotor J 4.2 at 3500 g for 30 min. Supernatant was transferred to a clean 4 L beaker and benzamidine and sodium azide were added to final concentrations of 1 mM and 0.01% (w/v), respectively. 50 ml of a concanavalin A Sepharose (ConA) resin was added to the supernatant and stirred at 200 rpm overnight. Using gravity flow, the resin was packed into a column and washed with five column volumes (CV) of TNC buffer. Protein was eluted with five column volumes of TNC buffer containing 0.6 M α-methyl mannose. Using a peristaltic pump, we loaded the eluate onto 11 ml protein A resin with immobilized antibody m159 [48] (10 mg per ml of protein A resin), specific for trimeric VP7, and equilibrated in buffer A (20 mM Tris, 50 mM NaCl, 0.1 mM CaCl_2_, pH 8.0). We washed bound VP7 protein with five CV of buffer, and eluted with five CV of buffer B (20 mM Tris, 50 mM NaCl, 1 mM EDTA, pH 8.0). Buffer was exchanged using a G25 PD10 column equilibrated in 0.1HNC (2 mM HEPES, 10 mM NaCl, 0.1 mM CaCl_2_) spiked with 0.1 mM PMSF. Protein was divided into aliquots, which were frozen in liquid nitrogen and stored at -80°C.

*Purification of recombinant wild type VP4 and S567C/A590C VP4 (“foot-locked” VP4)*–We harvested baculovirus carrying the genetic information of full-length wild type VP4 (P5B[3] serotype) or S567C/A590C VP4 from bacmid transfected Sf9 cells. Sf9 cells were then inoculated with baculovirus and passaged in the same cells three times, with an incubation time of 72 h for each passage. Four flasks of 500 ml Sf9 cells grown in suspension to a density of approximately 2 million cells per ml were then infected with 13 ml of passaged virus stock solutions and incubated for 72 h. Cells were harvested at 4°C by centrifugation in Beckman Coulter rotor J 4.2 at 3,500 g for 30 min. We discarded the supernatant, resuspended the cell pellet in 100 ml lysis buffer (75 mM Tris, 100 mM NaCl, 5 mM EDTA, 7.5% (v/v) glycerol, 1 mM PMSF, 1 mg/ml aprotinin, 1 mg/ml pepstatin, 1 mg/ml leupeptin), and froze the sample in liquid nitrogen for storage at -80°C. After thawing of the cell suspension, cells were lysed on ice with a Branson 450 Digital Sonifier (Branson Ultrasonics, Brookfield, CT) with 40% amplitude, for 5 min with 50% duty cycles. After 5 min incubation on ice, the sonification procedure was repeated. We the centrifuged the suspension 4°C in a Beckman Coulter rotor Ti 45 at 104,000 g for 1 h, collected the supernatant and precipitated VP4 by adding 0.244 g ammonium sulfate per ml of protein solution. The resulting suspension was stirred in a glass flask overnight at 4°C at 200 rpm and then spin at 4°C in Beckman Coulter rotor Ti45 for 30 min at 104,000 g. We discarded the supernatant, resuspended the pellet in 60 ml of TE buffer (20 mM Tris, 1 mM EDTA, pH 8.0) with 1 mM PMSF, and transferred the solution to a Dounce homogenizer for homogenization with 15 strokes. The suspension was then spun at 4°C for 30 min in a Beckman Coulter rotor Ti 45 at 104,000 g. Supernatant was collected and diluted with TE buffer with 1 mM PMSF to a final volume of 900 ml and loaded onto a 5 ml Q Sepharose column (Cytiva) using a peristaltic pump at a flow rate of 5 ml/min. Using an HPLC purification system, the column was washed with two CV of T10NE buffer (20 mM Tris, 10 mM NaCl, 1 mM EDTA, pH 8.0), and bound protein was eluted with 20 CV using a linear gradient from T10NE to T150NE (20 mM Tris, 150 mM NaCl, 1 mM EDTA, pH 8.0) at a flow rate of 1 ml/min. We pooled VP4-containing protein fractions, concentrated them to 500 μl using a 10 kDa MWCO centricon (Millipore Sigma), and exchanged the buffer to HNE with 0.1 mM PMSF using a G25 PD10 column. Protein was distributed to 420 μg aliquots and frozen in liquid nitrogen for storage at -80°C.

*Liposome preparation*– 2 mg in total lipids of a mixture containing cholesterol, phosphocholine egg extract (egg PC), sphingomyelin (SM), 1,2-dioleyl-sn-glycero-3-phopshoethenolamine (DOPE), 1-palmitoyl-2-oleyo-sn-glycerol-3-phosphoetanolamine (POPE), 1,2-dioleyl-sn-glycero-3-phospho-L-serine (DOPS) and ganglioside GD1a (GD1a) in molar ratio of 50:12.5:10:8.5:8.5:8:2.5, dissolved in approximately 9:1 chloroform:methanol, were dried in a round bottom glass tube under argon flow. Remaining solvent was completely removed by incubating the lipids under high vacuum overnight. The lipids were resuspended in 200 μl of TNC buffer by vortexing for two minutes, in a volume corresponding to 10 mg/ml. We then freeze-thawed the lipid suspension twice using liquid nitrogen and formed liposomes by extrusion though a 0.2 μm filter using 41 passages. We evaluated the size of liposomes by dynamic light scattering using a 1:100 dilution of the liposome stock.

*Recoating of DLPs with recombinant VP7 and VP4* –We distributed 45 μg of DLPs in HNE equally among five 1.5 ml conical tubes (2.25 μl per tube). We first added 1 M sodium acetate, pH 5.2 to a final concentration of 100 mM and then added 65 μl VP4 (stored at 1.3 mg/ml) to a final concentration of 0.9 mg/ml in the final reaction volume, resulting in a 33-fold excess of VP4 monomer over a total of 180 sites on DLPs. A 0.1 mg/ml aprotinin solution was added to the samples to a final concentration of 0.2 μg/ml followed by incubation at 37°C for 1 h. Required amounts of VP7 (9 μl stored at 1.0 mg/ml in HNE) to achieve a 2.3-fold excess of VP7 monomer over a total of 780 sites on DLPs were premixed with 0.1 volumes of TC buffer (20 mM Tris, 10 mM CaCl_2_, pH 8.0) and 0.1 volumes of 1 M sodium acetate pH 5.2 for 15 min before adding them to the DLP-VP4 mixture. Samples were incubated for 1 h at room temperature and then quenched by the addition of 0.1 volumes of 1 M Tris pH 8.0. Recoated particles from the five tubes were combined, and TNC was added to a final volume of 2.5 ml. rcTLPs were separated from excess VP4 and VP7 by ultracentrifugation at 4°C in a Beckman Coulter rotor TLS 55 at 215,000 g for 1 h. We removed 2.0 ml of the supernatant, returned the volume to 2.5 ml with TNC, and pelleted again. Supernatant was carefully removed so that 100 to 200 μl remained. The rcTLP pellets were resuspended in the remaining buffer, sodium azide added to a final concentration of 0.01%, and the rcTLPs stored at 4°C.

### Ca^2+^ permeabilization experiment and TIRF microscopy

*Labelling of TLPs and rcTLPs–*TLPs or rcTLPs (mutant or wt) were diluted to 1.0 mg/ml in a total volume of 30 μl using HNC (20 mM HEPES pH 8.0, 100 mM NaCl, 1 mM CaCl_2_) and 3.33 μl of 1 M NaHCO_3_ pH 8.3 added. This solution was mixed with 0.5 μl of 0.0076 mg/ml Atto 565 NHS ester. The reaction proceeded at room temperature for 1 h before quenching with 3 μl of 1 M Tris pH 8.0. The labeled TLPs were then buffer exchanged into a solution containing 20 mM Tris pH 8.0, 100 mM NaCl, and 1 mM CaCl_2_ using a Zeba Spin Desalting Column (Thermo Fisher Scientific).

*pH shift experiments with rcTLPs*–rcTLPs were incubated with 5 μg/ml trypsin for 30 min at 37°C and the reaction quenched by addition of 1 mg/ml aprotinin. The high-pH induced conformational change was achieved by adding 0.7 M N-cyclohexyl-3-aminopropanesulfonic acid (CAPS) buffer at pH 11.0 to a final concentration of 54 mM. Samples were incubated for 30 min at room temperature and neutralized to pH 8.0 by addition of diluted HCl. As a control, we used 20 mM Tris-HCl, 54 mM CAPS pH 8.0 to match the osmolarity of both buffers.

*Liposome preparation*–A chloroform solution containing 2 mg total lipid mixture of cholesterol, phosphocholine egg extract (egg PC), sphingomyelin (SM), 1,2-dioleyl-sn-glycero-3-phopshoethenolamine (DOPE), biotinylated-DOPE, Cy5-DOPE, 1-palmitoyl-2-oleyo-sn-glycerol-3-phosphoetanolamine (POPE), 1,2-dioleyl-sn-glycero-3-phospho-L-serine (DOPS) and ganglioside GD1a (GD1a) in molar ratio of (40:22.5:10:7.25:0.5:0.25:8.5:8:2.5) was dried in a round bottom glass tube under argon flow. Remaining solvent was removed by incubating the lipids under high vacuum overnight. We then resuspended the lipids in 200 μl of TNE (20 mM Tris pH 8.0, 100 mM NaCl, 0.07 mM EGTA) containing 400 μM Fluo-4 dye by vortexing for two minutes, forming a 10 mg/ml lipid suspension, which we subjected to two freeze-thaw cycles with liquid nitrogen. For the pH shift experiments, the lipids were resuspended in 200 μl of TNE (20 mM Tris-HCl, 100 mM NaCl, 0.07 mM EGTA), 54 mM CAPS, pH 8.0, containing 400 μM Fluo-4 dye, so that the buffer had the same osmolarity that the final preparation of rcTLPs. By including 0.07 mM EGTA and then exchanging the liposomes into a buffer containing 1 mM CaCl_2_ before incubating them with TLPs (see below), we ensured that there was no free Ca^2+^ inside the liposomes. Liposomes were formed by extrusion with 41 passages through a 200 nm pore filter. The size of the liposomes was evaluated with dynamic light scattering using a 1:100 dilution of the liposome stock. The liposome suspension was loaded onto a G25 PD10 column equilibrated with TNC (or TNC, 54 mM CAPS pH 8.0 in the case of pH shift experiments) to separate liposomes from non-incorporated fluorophore and for buffer exchange. We collected 500 μl fractions and used peak fractions containing liposomes for further experiments.

*Liposome Ca*^*2+*^ *permeabilization assay–* 4 μl of liposomes were added to 12 μg virus particles in a final volume of 18 μl of TNC (or TNC, 54 mM CAPS pH 8.0) and incubated for 30 min at 37°C with 5 μg/ml trypsin (to activate VP4). Trypsin was inactivated by adding aprotinin to a final concentration of 1 mg/ml. All samples were incubated for an additional 1 h at 37°C. For controls, same amount of liposomes was incubated for the same time and temperature in the absence of virus (negative control) or in the presence of 20 μM ionomycin (positive control).

*TIRF measurements*– 25 mm circular glass coverslips were cleaned and coated with a 10% (w/v) solution of biotinylated-polyethylene glycol (PEG):PEG in a ratio 1:99 (Laysan Bio, cat. no. mPEG-SCM-5000) [49] and pre-incubated with 0.5 mg/ml neutrAvidin (Thermo Fisher Scientific, cat. no. LF144746) for 15 min at room temperature (RT) before use. Liposome solutions were diluted 500 times in TNC in order to avoid stochastic co-localizations of virus and liposomes, incubated over the coverslip for 10 min at RT, and unbound liposomes were removed before imaging by a single wash with 100 μl TNC. Images of different fields of view were acquired under TIRF illumination using an Olympus IX70 microscope equipped with a Hamamatsu ImageEM camera and excitation from 488, 561 and 640 nm lasers (Coherent, Inc., Salem, NH) operated at 100, 150 and 70 mW, respectively (data from Figs 2, S1 and S2). For each field of view, we recorded three images with 50% power from each laser and 30 ms exposure at 488 nm (Fluo-4 dye, Ca^2+^ sensor signal), 50 ms exposure at 561 nm (Atto 565 NHS ester dye, virus signal), 20 ms exposure at 640 nm (Cy5-DOPE dye, liposome signal). A Zeiss 200M microscope equipped with a Zeiss TIRF slider, a Photometrics Cascade camera and 488, 561 and 640 nm excitation lasers (Coherent, Cobalt and Coherent, Inc., respectively) operated at 100 mW, 50 mW and 40 mW, respectively, was also used for acquiring additional images (data from Figs 3, S3, S5, S6 and S7). The microscopes were controlled by the SlideBook acquisition program (Intelligent Imaging Innovations, Denver, CO), and image processing was carried out using Fiji software [50].

*Infectivity assay of TLPs and rcTLPs–*Particles to focus-forming unit (FFU) ratios for TLP and rcTLPs were determined by infectious focus assays as previously described [27], and specific infectivities were calculated from concentration measurements on the basis of densitometry of a coomassie-blue stained SDS-PAGE gel. BSC-1 cells were washed with Gibco M199 media (Thermo Fisher Scientific), inoculated with serial dilutions of virus in M199 containing 1 μg/ml trypsin and incubated for 1 h at 37°C. The inoculum was removed and replaced with M199 containing 10% fetal bovine serum and 2.5 μg/ml neutralizing monoclonal antibody M159 to inhibit secondary infection. At 16 h postinfection, cells were washed with phosphate-buffered saline and fixed with methanol. Infectious foci detected by immunoperoxidase staining were counted. Monoclonal antibody M60, which recognizes VP7, was used as the primary detection antibody.

*Image analysis*–We used previously described custom-made MATLAB (MathWorks) scripts [51] for automatic detection of fluorescent liposomes in the 640 nm channel (Cy5-DOPE dye, liposome signal) images by fitting a 2D Gaussian function to diffraction limited spots for which the intensity was 1.5 above background. We extracted the XY position of each liposome in all images and calculated the integrated fluorescence intensity on a 4x4 pixel area around the XY position for the 3 channels. We calculated the background by measuring the integrated fluorescence intensity on a 6x6 pixel region around the XY position, which was then was subtracted from the integrated intensity on the 4x4 area after area correction for the 3 different channels. The integrated fluorescence intensity after background correction is shown in the figures as “Ca^2+^ signal” and “liposome signal” (arbitrary units, AU). For the samples that were incubated with TLPs and rcTLPs, the liposomes that colocalized with virus were selected by applying a threshold to the integrated fluorescence intensity in the 561 channel (Atto 565 NHS ester dye, virus signal) at the specific XY position of the liposomes; the threshold was calculated from images, taken at the same time, that contained only virus.

### Cryo-EM specimen preparation and data collection

*Incubation of rcTLPs with liposomes*–To rcTLP stock solutions (0.7–1.5 mg/ml), we added liposome stock solutions (10 mg/ml, average diameter 200 nm) to a final concentration of 1.5 mg/ml, followed by the addition of trypsin to a final concentration of 5 μg/ml and incubation for 30 min at 37°C. Trypsin was then inactivated with aprotinin at a final concentration of 1 mg/ml. Samples were incubated at 37°C for an additional 30 min.

*Cryo-EM sample preparation*–C-flat holey copper carbon grids (CF-2/1-2C, Electron Microscopy Sciences) were glow discharged for 30 s at 10 mA. Using a CP3 cryo-plunger, 4 μl of virus-liposome samples were applied to the grids, blotted for 4 s with at a humidity ranging from 88% to 92% and plunged into liquid ethane having a temperature of -273°C. Grids were stored in liquid nitrogen.

*Cryo-EM data collection*–We recorded movies with a Titan Krios G3i microscope (Thermo Fisher Scientific, Waltham, MA), operated at 300 kV and equipped with a BioQuantum energy filter and a K3 direct electron detector camera (Gatan, Inc.). Dose-fractionated movies with 50 frames were recorded in counting mode with 0.05 s per frame for a total of 2.5 s with SerialEM v3.7 [52]. The magnification was 60,606, resulting in a pixel size of 0.825 Å per pixel. For some grids, we recorded a total of 27 movies for each stage position (nanoprobe mode with an illuminated area of ~1 μm, 3 exposures per hole from a total of 9 holes).

### Cryo-EM data processing

*Preprocessing and initial particle stack preparation*–We used MotionCor2 [53] with 5×5 patch alignment to obtain summed micrographs from the frames of 33,563 movies that we had recorded from four sample preparations. With CTFFIND4 [54], we estimated contrast transfer function (CTF) parameters from the summed micrographs. We then picked 77,139 rotavirus particles from the micrographs template matching in cisTEM [29,30], with the routines match_template (version 1.00), refine_template (version 1.00), and make_template_result (version 1.00). We carried out template matching at 5 Å resolution with angular search steps of 1.7° and a previously determined reconstruction as reference structure [18], resampled to the correct pixel size. The symmetry for the search was I_2_ and the radius of the mask 403 Å. We determined CTF parameter values at particle positions with Gctf [55] and used relion_preprocess [56] for particle extraction and normalization. The box size was 1536×1536 pixels and the radius for background area designation 666 pixels. After 2D classification with relion_refine [56], we kept 75,846 particle images for analysis.

*Icosahedral reconstruction*–An initial reconstruction calculated with the alignment parameters obtained from template matching had a nominal resolution of 3.89 Å as determined by Fourier shell correlation (FSC) with a cutoff value of 0.143 and after masking the half maps with a spherical shell mask that had inner and outer radii of 222 and 403 Å, respectively (S9 Fig). We then improved particle alignment, particle images, and reconstruction parameters by iterating routines in cisTEM (refine3d version 1.01, reconstruct3d version 1.02) [29] with routines in RELION (relion_motion_refine, and relion_ctf_refine) [57]. The resolution for alignment in the last iteration was 3.4 Å. In relion_ctf_refine, we used per-particle defocus fitting, beam-tilt refinement, symmetric aberration estimation, and fitting of anisotropic magnification distortion for each of the particle stack’s 63 optics groups (—fit_defocus–kmin_defocus 30 –fit_mode fpmfp–fit_beamtilt–kmin_tilt 30 –fit_aniso–odd_aberr_max_n 3 –fit_aberr). Reconstuctions were calculated with relion_reconstruct with the option–fom_weighting, where we assigned a per particle figure of merit, *FOM*, based on the cisTEM alignment scores, where *S*_*i*_ is the score of particle *I*, and *S*_*max*_ and *S*_*min*_ are the highest and lowest scores, respectively:

$$FOM=\frac{S_{i}-S_{min}}{S_{max}-S_{min}}$$

After two iterations, the nominal resolution of the final icosahedral reconstruction of full viral particles converged to 2.43 Å as estimated with relion_postprocess (S9 Fig and S1 Table).

*Local analysis of spike positions*–For local analysis of individual spike positions, we used protocols similar to those previously described [10,18,37], with some modifications (S10 Fig). Generally the workflow for preparing subparticle stacks for local analysis involved symmetry expansion of the RELION particle star file, followed by signal subtraction with relion_project using a suitable mask to retain density, and sub-particle extraction from the signal-subtracted images with custom Python scripts and IMOD routines [58]. Depending on the analysis, different subparticle stacks were prepared.

For initial classification of spike positions—that is, determining for each of the 60 positions on the virion whether the VP5*/VP8* penetration protein was in an upright, intermediate or reversed conformation, or whether it was entirely absent—we made a 384×384 pixel subparticle stack 1 in which we had signal-subtracted all density except within the volume of a single VP7 trimer plus the volume potentially occupied by VP5*/VP8* in upright and reversed conformations (S10A–S10D Fig). We carried out classifications with refine3d and reconstruct3d from cisTEM [29], keeping the subparticle alignment angles and shifts constant during all iterations. We also turned off per-particle weighting in the reconstruction step by setting the particle weighting factor input parameter of reconstruct3d to 0.0 and by resetting the LogP, SIGMA, and SCORE values in par files to constant values of -2000, 4.1, and 20.1, respectively, and running the reconstruction step again before proceeding to the next iteration. Initial classes were seeded randomly. In a first classification (classification #1), we asked for 16 classes (S2 Table). The resolution limit for classification was 5 Å and the radius of the spherical mask applied to the 2D images was 140 Å. 3D references were masked around the single VP7 trimer and VP5*/VP8* volume. Inspection of the resulting classes from the first classification showed that particles partitioned not only based on spike occupancy and conformation, but also based on radial shifts (up to approximately 5 Å) with respect to the virion center (S1 Movie). As evident from S11 Fig, the main cause for the observed radial shifts appeared to be anisotropic magnification distortion, because the class assignment of subparticles from the two classes with the largest relative shift observed (classes 4 and 13) showed a systematic bias depending on the extraction angle with respect to the virion center. We corrected for anisotropic magnification when we calculated icosahedral reconstructions of full viral particles, but no correction was implemented during subparticle extraction. Other imperfections probably also contributed to the observed shifts; for instance, if data had been collected slightly away from the eucentric height of the microscope. We therefore further classified each of the 16 classes in a second classification (classification #2), aligned all 32 classes by rigid-body fitting a model of the VP7 trimer and updating the alignment parameters in the RELION star file based on the superpositions of the fitted models, and made a final assignment of each subparticle as upright, intermediate, reversed, or unoccupied (S10E, S10F and S12 Figs and S2 Table).

Local analysis of reversed VP5*/VP8* spikes bound to liposomes–Because we observed minor density artifacts in reconstructions calculated from subparticle stack 1 (see above) when contoured at very low level (presumably caused by a bug or array overflow in relion_project when the volume for signal subtraction had a large size of 1536×1536×1536 pixels), we prepared subparticle stack 2 with slightly down sampled data of 1024×1024×1024 pixels for the full virus particle box, corresponding to a pixel size of 1.2375 Å. For this stack, we signal-subtracted all density within a tight-fitting spherical mask except within the volume occupied by VP5*/VP8* at the reference icosahedral position (S10C and S10D Fig). Classifications #3 and #4 were carried out starting with 1,510,279 particles in reversed conformation selected from the signal-subtracted subparticle stack 2 based on the results from classification #1 and #2 (S10G Fig). 2D and 3D masking was with a radius of 124 Å, and the resolution for classification was 5 and 8 Å, respectively. Subparticles from classes 1 and 3 of classification #3 that showed liposome density were merged and subjected to classification #4 (S13 Fig and S3 Table). Final reconstructions of liposome-bound classes were computed from the original (no signal subtraction) subparticle stack 2 (S10H Fig).

*Mapping of subparticles in the original micrographs*–When we extracted subparticles, we assigned unique viral particle and subparticle identifiers together with the *x* and y coordinates of the extraction position of each subparticle with respect to the original extraction position of the corresponding virus in the micrograph. We could thus map subparticles from liposome-bound classes onto the original micrographs (Fig 4D).

### Figure preparation

We prepared the figures using PyMOL (The PyMOL Molecular Graphics System, Version 2.3 Schrödinger, LLC), ChimeraX [59], BioRender, matplotlib [60] and ImageMagick (ImageMagick Studio LLC, 2023, available at: https://imagemagick.org).

## Supporting information

S1 Fig Single-particle fluorescence imaging. (A) Fluorescence intensity for each liposome in three independent experiments (Experiment 1, 2 and 3). Fluorescence intensity corresponding to the control sample (left), the ionophore sample (center) or TLP sample (right) are shown for each experiment, respectively. Top graph (cyan) shows the signal in the 640 nm channel (Cy5-DOPE dye, liposome signal); center graph (green) shows the signal in the 488 nm channel (Fluo-4 dye, Ca^2+^ sensor signal); and bottom graph (magenta) shows the signal in 561 nm channel (Atto 565 dye, TLP signal). (B) Normalized frequency of the liposome signal for the control sample (white bars), ionophore sample (light grey bars) and TLP sample (all liposomes, dark grey bars) in each experiment.
(TIF)

S2 Fig Ca^2+^-signal analysis from single-particle fluorescence imaging. (A) Ca^2+^ signal intensity (arbitrary units, AU) as a function of liposome signal intensity (AU) for the control sample (white dots) and the ionophore sample (grey dots) in experiments 1, 2 and 3, respectively. (B) Ca^2+^ signal intensity as a function of the binned liposome signal for the control sample (white bars) and the ionophore sample (grey bars) in experiments 1, 2 and 3, respectively. Error bars represent the standard deviation calculated from individual measurements within each bin as shown in the scatter plot in panel A. (C) Ca^2+^ signal intensity as a function of liposome signal intensity for internal control (small random selection of liposomes that did not colocalize with virus in the TLP sample, white dots) and for TLP (liposomes colocalizing with virus only, grey dots) samples in experiments 1, 2 and 3, respectively. (D) Ca^2+^ signal as a function of the binned liposome signal for the internal control (liposomes that do not colocalize with virus in the TLP sample, hatched white bars) and the TLP sample (liposomes colocalizing with virus only, hatched grey bars) in experiments 1, 2 and 3, respectively. Error bars represent the standard deviation calculated from individual measurements within each bin as shown in the scatter plot in panel C. Statistical analysis in B and D used a t-test between samples. n.s. = p>0.05; * = p<0.05; ** = p<0.01; *** = p<0.001.
(TIF)

S3 Fig Ca^2+^-signal analysis for TLP virus and wt rcTLP virus from single-particle fluorescence imaging. (A) Ca^2+^ signal intensity (arbitrary units, AU) as a function of liposome signal intensity (AU) for the control sample (white dots) and for ionophore sample (grey dots) in experiments 1, 2 and 3, respectively. (B) Ca^2+^ signal intensity as a function of the binned liposome signal intensity for the control sample (white bars) and the ionophore sample (grey bars) in experiments 1, 2 and 3, respectively. Error bars represent the standard deviation calculated from individual measurements within each bin as shown in the scatter plot in panel A. (C) Ca^2+^ signal intensity as a function of liposome signal intensity for the internal control in the wt rcTLP sample (random selection of liposomes that do not colocalize with virus in the wt rcTLP sample, white dots) and for wt rcTLP (liposomes colocalizing with wt rcTLP only, grey dots) in experiments 1, 2, 3, 4, and 5, respectively. (D) Ca^2+^ signal intensity as a function of the binned liposome signal for the internal control in the wt rcTLP sample (liposomes that do not colocalize with virus in the TLP sample, dotted white bars) and wt rcTLP (liposomes colocalizing with virus only, dotted grey bars) in experiments 1, 2, 3, 4, and 5, respectively. Error bars represent the standard deviation calculated from individual measurements within each bin as shown in the scatter plot in panel C. Statistical analysis was performed in B and D using a t-test between samples. n.s. = p>0.05; * = p<0.05; ** = p<0.01; *** = p<0.001.
(TIF)

S4 Fig SDS-PAGE gel and infectivity of TLP, wt rcTLP and mut rcTLP. (A) Coomassie-blue stained SDS-PAGE gel of the mut rcTLP (lane 1), TLP (lane 2) and wt rcTLP (lane 3) samples. A quantitative analysis of band intensity, using Fiji [50] and normalizing by the VP6 band intensity, estimated that the wt rcTLPs had only ~25% of theVP5* present in TLPs from cells and that the mut rcTLPs had ~30% more. Wild-type recoating varies considerably from preparation to preparation; the one used here had relatively low occupancy. (B) Particle to focus-forming unit (P/FFU) ratios for mut rcTLP, TLP and wt rcTLP samples. The bar represents the average for 2 measurements and error bars represent the standard deviation.
(TIF)

S5 Fig Ca^2+^-signal analysis for wt rcTLP virus incubated at pH 8.0 or pH 11.0 from single-particle fluorescence imaging. (A) Ca^2+^ signal intensity (arbitrary units, AU) as a function of liposome signal intensity (AU) for the internal control in the wt rcTLP sample (random selection of liposomes that did not colocalize with virus in the wt rcTLP sample, white dots) and for wt rcTLP (liposomes colocalizing with virus only, grey dots) pre-incubated at pH 8.0, in experiments 1, 2 and 3, respectively. (B) Ca^2+^ signal intensity (AU) as a function of the binned liposome signal for the internal control in the wt rcTLP sample (dotted white bars) and for wt rcTLP (dotted grey bars) pre-incubated at pH 8.0, in experiments 1, 2 and 3, respectively. Error bars represent the standard deviation calculated from individual measurements within each bin as shown in the scatter plot in panel A. (C) Ca^2+^ signal intensity (AU) as a function of liposome signal for the internal control in the wt rcTLP sample (random selection of liposomes that did not colocalize with virus in the wt rcTLP sample, white dots) and for wt rcTLP (liposomes colocalizing with virus only, grey dots) pre-incubated at pH 11.0, in experiments 1, 2 and 3, respectively. (D) Ca^2+^ signal intensity (AU) as a function of the binned liposome signal for the control in the wt rcTLP sample (dotted white bars) and for wt rcTLP (dotted grey bars) pre-incubated at pH 11.0, in experiments 1, 2, and 3, respectively. Error bars represent the standard deviation calculated from individual measurements within each bin as shown in the scatter plot in panel C. Statistical analysis was performed in B and D using a t-test between samples. n.s. = p>0.05; * = p<0.05; ** = p<0.01; *** = p<0.001.
(TIF)

S6 Fig Ca^2+^-signal analysis for mutant rcTLP virus incubated at pH 8.0 or pH 11.0 from single-particle fluorescence imaging. (A) Ca^2+^ signal intensity (arbitrary units, AU) as a function of liposome signal intensity (AU) for the internal control in the mutant rcTLP sample (random selection of liposomes that did not colocalize with virus in the mutant rcTLP sample: white dots) and for mutant rcTLP (liposomes colocalizing with virus only, grey dots) pre-incubated at pH 8.0, in experiments 1, 2 and 3, respectively. (B) Ca^2+^ signal intensity as a function of the binned liposome signal for the internal control in the mutant rcTLP sample (cross hatched white bars) and the mutant rcTLP (cross hatched grey bars) pre-incubated at pH 8.0, in experiments 1, 2 and 3, respectively. Error bars represent the standard deviation calculated from individual measurements within each bin as shown in the scatter plot in panel A. (C) Ca^2+^ signal intensity (AU) as a function of liposome signal for the internal control in the mutant rcTLP sample (random selection of liposomes that did not colocalize with virus in the mutant rcTLP sample: dots in white bar) and for mutant rcTLP (liposomes colocalizing with virus only, dots in gray bar) pre-incubated at pH 11.0, in experiments 1, 2 and 3, respectively. (D) Ca^2+^ signal intensity as a function of the binned liposome signal for the mutant rcTLP internal control (cross hatched white bars) and the mutant rcTLP sample (liposomes colocalizing with virus only, cross hatched grey bars) pre-incubated at pH 11.0, in experiments 1, 2 and 3, respectively. Error bars represent the standard deviation calculated from individual measurements within each bin as shown in the scatter plot in panel C. Statistical analysis was performed in B and D using a t-test between samples. n.s. = p>0.05; * = p<0.05; ** = p<0.01; *** = p<0.001.
(TIF)

S7 Fig Brief treatment of rotavirus particles at pH 11 before liposome incubation prevents Ca^2+^ permeabilization. (A) Percent Ca^2+^-positive liposomes for control liposomes (white bars), ionophore-exposed liposomes (grey bars), internal control in the wt rcTLP sample (liposomes in the wt rcTLP sample that did not colocalize with wt rcTLP: dotted white bars) and wt rcTLP sample (liposomes that colocalized with wt rcTLP: dotted grey bars) from experiments 1, 2, 3, 4 and 5, respectively (S3 Fig). (B) Percent Ca^2+^-positive liposomes for the internal control in the wt rcTLP sample and wt rcTLP pre-incubated at pH 8.0 (dotted white and dotted grey bars, respectively), and for the internal control in the wt rcTLP sample and wt rcTLP pre-incubated at pH 11.0 (dotted white and dotted grey bars on grey background, respectively) from experiments 1, 2 and 3, respectively (S5 Fig). (C) Ca^2+^-positive liposomes for the internal control in the mutant rcTLP sample and mutant rcTLP pre-incubated at pH 8.0 (crossed hatched white and crossed hatched grey bars, respectively), and for the internal control in the mutant rcTLP sample and mutant rcTLP pre-incubated at pH 11.0 samples (cross hatched white and cross hatched grey bars on grey background, respectively) from experiments 1, 2 and 3, respectively (S6 Fig). "Ca^2+^-positive liposomes" designates the percent of toal liposomes scored that had a Ca^2+^ signal intensity greater than 1.5 standard deviations from the mean in the corresponding control sample (see red dashed lines in S3A, S3C, S5A, S5C, S6A and S6C Figs for the cutoffs).
(TIF)

S8 Fig Cryo-EM sample preparation of liposome-bound rotaviruses. Schematic illustration of the interaction between rotavirus and a liposome during specimen preparation for cryo-EM analysis.
(TIF)

S9 Fig Icosahedral reconstruction of RRV particles. (A) Fourier shell correlations (FSCs) between half maps calculated from icosahedral reconstructions after applying a spherical shell mask encompassing the three protein layers of the TLP (VP2, VP6, and VP7). Curves are shown for the data processing steps as described in Methods. The final nominal resolution was 2.43 Å. (B) Local resolution analysis of the icosahedral reconstruction. A segment of the “triple-layer” particle is shown. (C) Density maps of VP2, VP6 and VP7 regions.
(TIF)

S10 Fig Cryo-EM analysis processing scheme of TLPs with liposomes. (A) Alignment of full viral particles with icosahedral symmetry imposed. A map of the final icosahedral reconstruction is shown where the particle is partially cut. VP1 (RNA-dependent RNA polymerase) and RNAs are colored gray; VP2, cyan and blue; VP6, green; VP7, yellow. See also S9 Fig. (B) Icosahedral symmetry expansion (60-fold) based on the full particle alignment. (C) Signal subtraction. The masks used to the define the subtraction volume, excluding the region of interest at a single protomer position, are shown. Signal-subtracted stack 1 was used to prepare subparticles for classifications #1 and #2 (spike conformation and occupancy). Signal-subtracted stack 2 was used to prepare subparticles for classifications #3 and #4 (liposome interaction of reversed spikes). (D) Subparticle extraction from the signal-subtracted and original particles stacks. (E) Classifications #1 and #2, see S12 Fig. (F) Alignment of classes to correct for observed shifts in reconstructions of subparticle classes caused primarily by anisotropic magnification distortion, see S11 Fig. Subparticle reconstructions are shown for the three observed spike conformations (upright, intermediate, and reversed) and empty positions. Density maps were low pass filtered at 5 Å resolution and partially cut. VP2, cyan and blue; VP6, green; VP7, yellow; VP5*, red, orange, and salmon. (G) Classifications #3 and #4, see S13 Fig. (H) Final reconstructions of liposome-bound classes calculated from subparticle stack 2, which was obtained from the original (non-signal-subtracted) images.
(TIF)

S11 Fig Observed subparticle shifts caused by anisotropic magnification distortion. (A) Subparticles were classified without alignment. Relative shifts between classes were determined by rigid-body fitting a V7 trimer model and then aligning the classes by updating the subparticle alignment parameters based on the fitted models. (B) Ribbon representation of the VP7 trimer models after fitting it into the densities of the 32 classes. The largest shift we observed was 5.7 Å between classes 4 and 13. (C) Each subparticle is associated with an extraction radius and angle with respect to the center of the full virus particle projection in the original micrograph. (D) Histograms of metadata values (extraction radius, extraction angle, defocus, optics group number, micrograph number) for all subparticles from the two classes 4 and 13, which showed the largest relative shift in their reconstructions. A strong bias in the extraction angle suggests that the observed shifts are predominately caused by anisotropic magnification distortion, which was not accounted for during subparticle extraction. (D) Histograms of metadata values (extraction radius, extraction angle, defocus, optics group number, micrograph number) for dataset 2 subparticles from the two classes 4 and 13.
(TIF)

S12 Fig Classification #1 and #2. For display, density maps were calculated from subparticle stack 1, which was obtained from the original (non-signal-subtracted) images, low pass filtered at 5 Å resolution and partially cut. VP2, cyan and blue; VP6, green; VP7, yellow; VP5*, red, orange, and salmon. (A) Classification #1 partitioned the particles into 16 classes, each of which was further subclassified into two classes in classification #2 (indicated by dashed lines). (B) Reconstructions of three observed spike conformations (upright, intermediate, and reversed) and empty positions after merging corresponding classes (S2 Table) and correcting of observed subparticle shifts (S11 Fig).
(TIF)

S13 Fig Classification #3 and #4. Classification of liposome-bound spike positions from subparticles with reversed spike conformations. (A) In classification #3, we initially requested four classes. Density maps were calculated from signal-subtracted subparticle stack 2, low pass filtered at 8 Å resolution and displayed in gray and at very low contour level to visualize membrane density. (B) In classification #4, we further subclassified classes 1 and 3 from classification #3. Density maps of the six classes were calculated from subparticle stack 2, which was obtained from the original (non-signal-subtracted) images, and low pass filtered at 8 Å. VP6, green; VP7, yellow; VP5*, red, orange, and salmon; membrane, gray.
(TIF)

S14 Fig Local resolution analysis of liposome-bound VP5*. (A) Full view of the class 5 and class 6 reconstructions colored according to local resolution. (B) Corresponding slab views.
(TIF)

S1 Table Cryo-EM data collection and statistics. (PDF)

S2 Table Classification #1 and #2 of VP5*/VP8* spike positions. (PDF)

S3 Table Classification #3 and #4 of reversed VP5*/VP8* spikes. (PDF)

S4 Table VP5*/VP8* spike conformations in liposome-bound versus liposome-unbound rcTLPs. (PDF)

S1 Movie Animation (of spike conformations). (GIF)
