# Supplementary figures and images for: The rotavirus VP5*/VP8* conformational transition permeabilizes membranes to Ca2+
Source: PLoS Pathog. 2024 Apr 4;20(4):e1011750. doi: 10.1371/journal.ppat.1011750 (PMC11020617; doi:10.1371/journal.ppat.1011750)

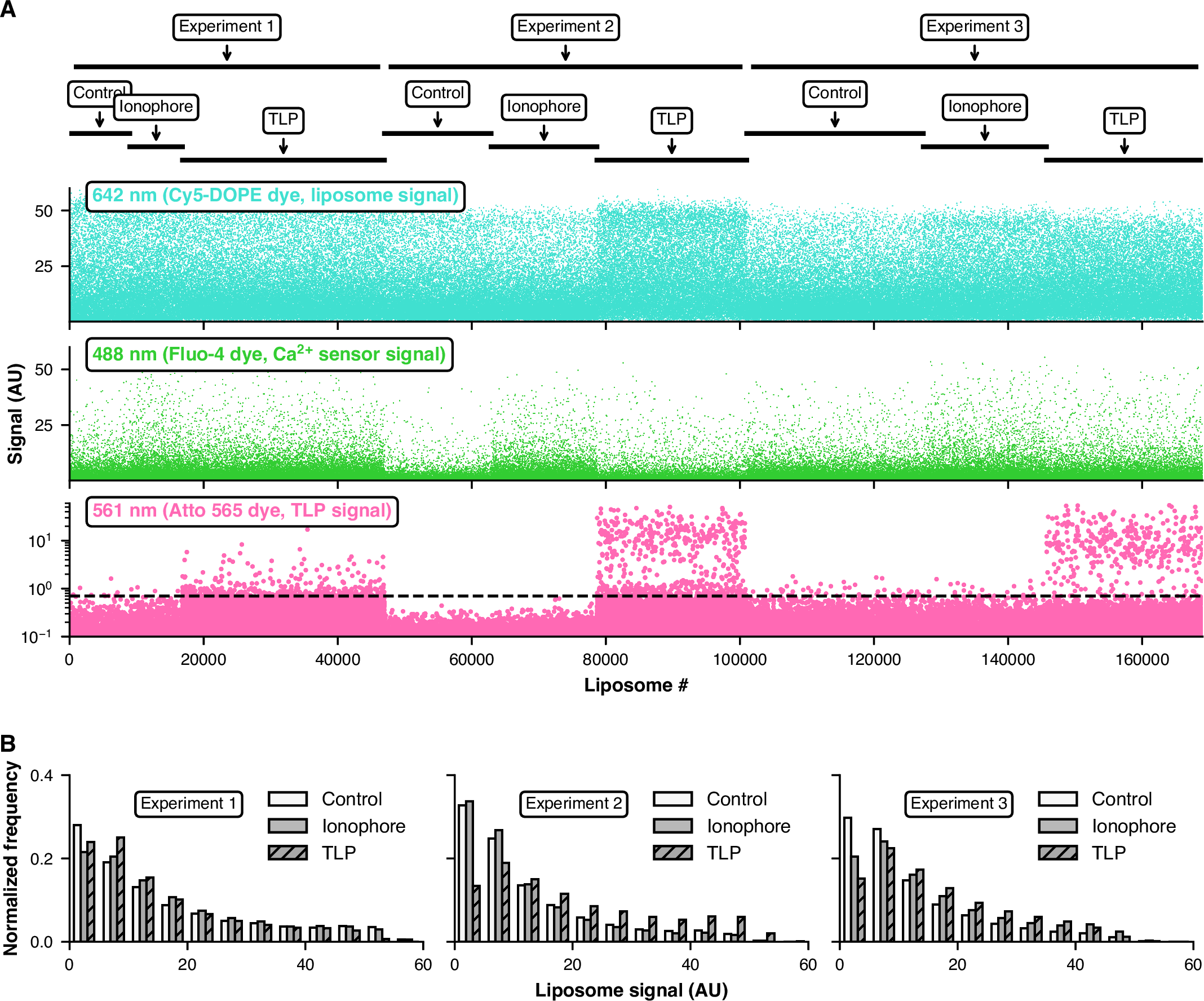

Supplement: S1 Fig — (A) Fluorescence intensity for each liposome in three independent experiments (Experiment 1, 2 and 3). Fluorescence intensity corresponding to the control sample (left), the ionophore sample (center) or TLP sample (right) are shown for each experiment, respectively. Top graph (cyan) shows the signal in the 640 nm channel (Cy5-DOPE dye, liposome signal); center graph (green) shows the signal in the 488 nm channel (Fluo-4 dye, Ca2+ sensor signal); and bottom graph (magenta) shows the signal in 561 nm channel (Atto 565 dye, TLP signal). (B) Normalized frequency of the liposome signal for the control sample (white bars), ionophore sample (light grey bars) and TLP sample (all liposomes, dark grey bars) in each experiment. (TIF) [file ppat.1011750.s001.tif]

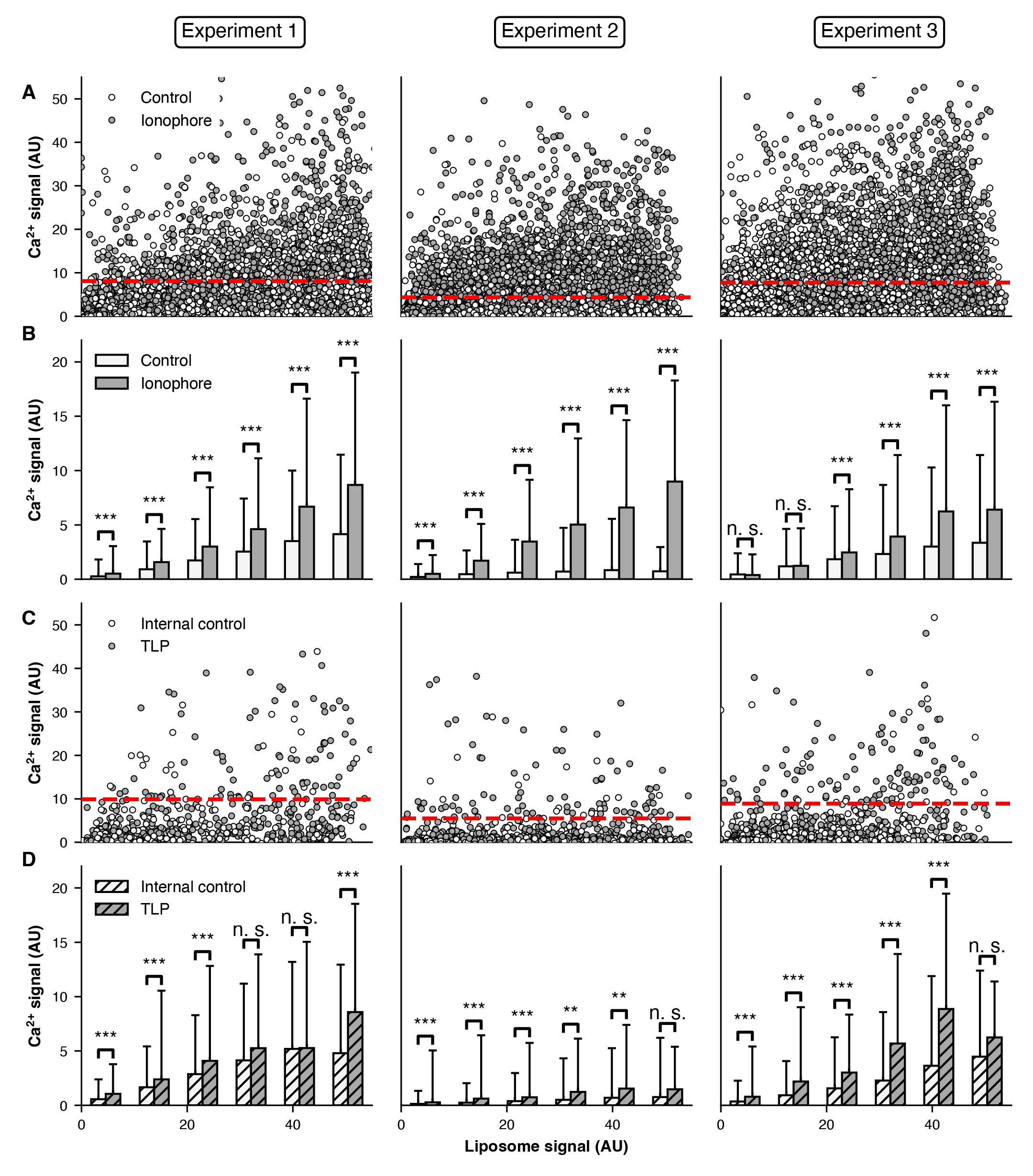

Supplement: S2 Fig — (A) Ca2+ signal intensity (arbitrary units, AU) as a function of liposome signal intensity (AU) for the control sample (white dots) and the ionophore sample (grey dots) in experiments 1, 2 and 3, respectively. (B) Ca2+ signal intensity as a function of the binned liposome signal for the control sample (white bars) and the ionophore sample (grey bars) in experiments 1, 2 and 3, respectively. Error bars represent the standard deviation calculated from individual measurements within each bin as shown in the scatter plot in panel A. (C) Ca2+ signal intensity as a function of liposome signal intensity for internal control (small random selection of liposomes that did not colocalize with virus in the TLP sample, white dots) and for TLP (liposomes colocalizing with virus only, grey dots) samples in experiments 1, 2 and 3, respectively. (D) Ca2+ signal as a function of the binned liposome signal for the internal control (liposomes that do not colocalize with virus in the TLP sample, hatched white bars) and the TLP sample (liposomes colocalizing with virus only, hatched grey bars) in experiments 1, 2 and 3, respectively. Error bars represent the standard deviation calculated from individual measurements within each bin as shown in the scatter plot in panel C. Statistical analysis in B and D used a t-test between samples. n.s. = p>0.05; * = p<0.05; ** = p<0.01; *** = p<0.001. (TIF) [file ppat.1011750.s002.tif]

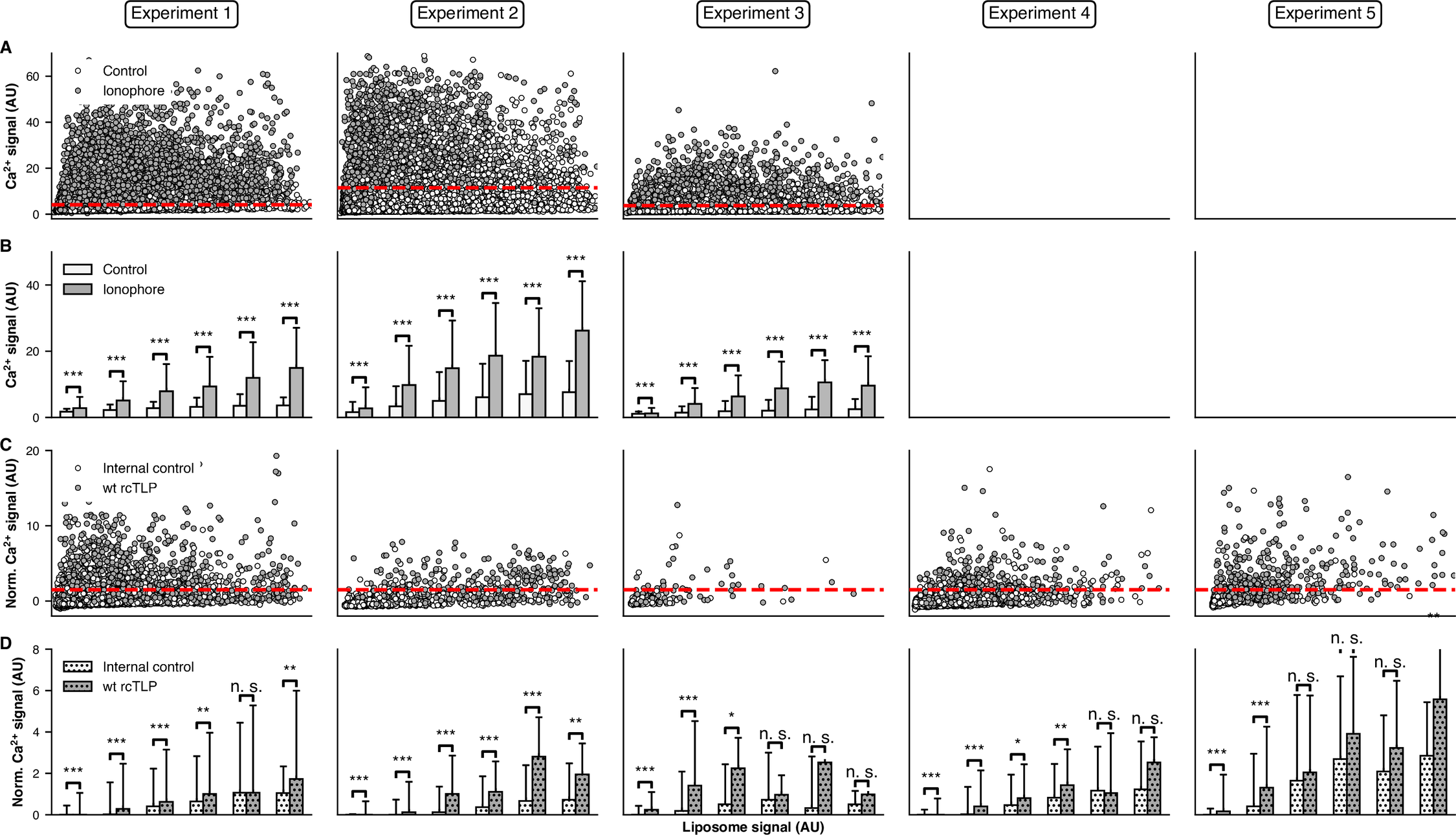

Supplement: S3 Fig — (A) Ca2+ signal intensity (arbitrary units, AU) as a function of liposome signal intensity (AU) for the control sample (white dots) and for ionophore sample (grey dots) in experiments 1, 2 and 3, respectively. (B) Ca2+ signal intensity as a function of the binned liposome signal intensity for the control sample (white bars) and the ionophore sample (grey bars) in experiments 1, 2 and 3, respectively. Error bars represent the standard deviation calculated from individual measurements within each bin as shown in the scatter plot in panel A. (C) Ca2+ signal intensity as a function of liposome signal intensity for the internal control in the wt rcTLP sample (random selection of liposomes that do not colocalize with virus in the wt rcTLP sample, white dots) and for wt rcTLP (liposomes colocalizing with wt rcTLP only, grey dots) in experiments 1, 2, 3, 4, and 5, respectively. (D) Ca2+ signal intensity as a function of the binned liposome signal for the internal control in the wt rcTLP sample (liposomes that do not colocalize with virus in the TLP sample, dotted white bars) and wt rcTLP (liposomes colocalizing with virus only, dotted grey bars) in experiments 1, 2, 3, 4, and 5, respectively. Error bars represent the standard deviation calculated from individual measurements within each bin as shown in the scatter plot in panel C. Statistical analysis was performed in B and D using a t-test between samples. n.s. = p>0.05; * = p<0.05; ** = p<0.01; *** = p<0.001. (TIF) [file ppat.1011750.s003.tif]

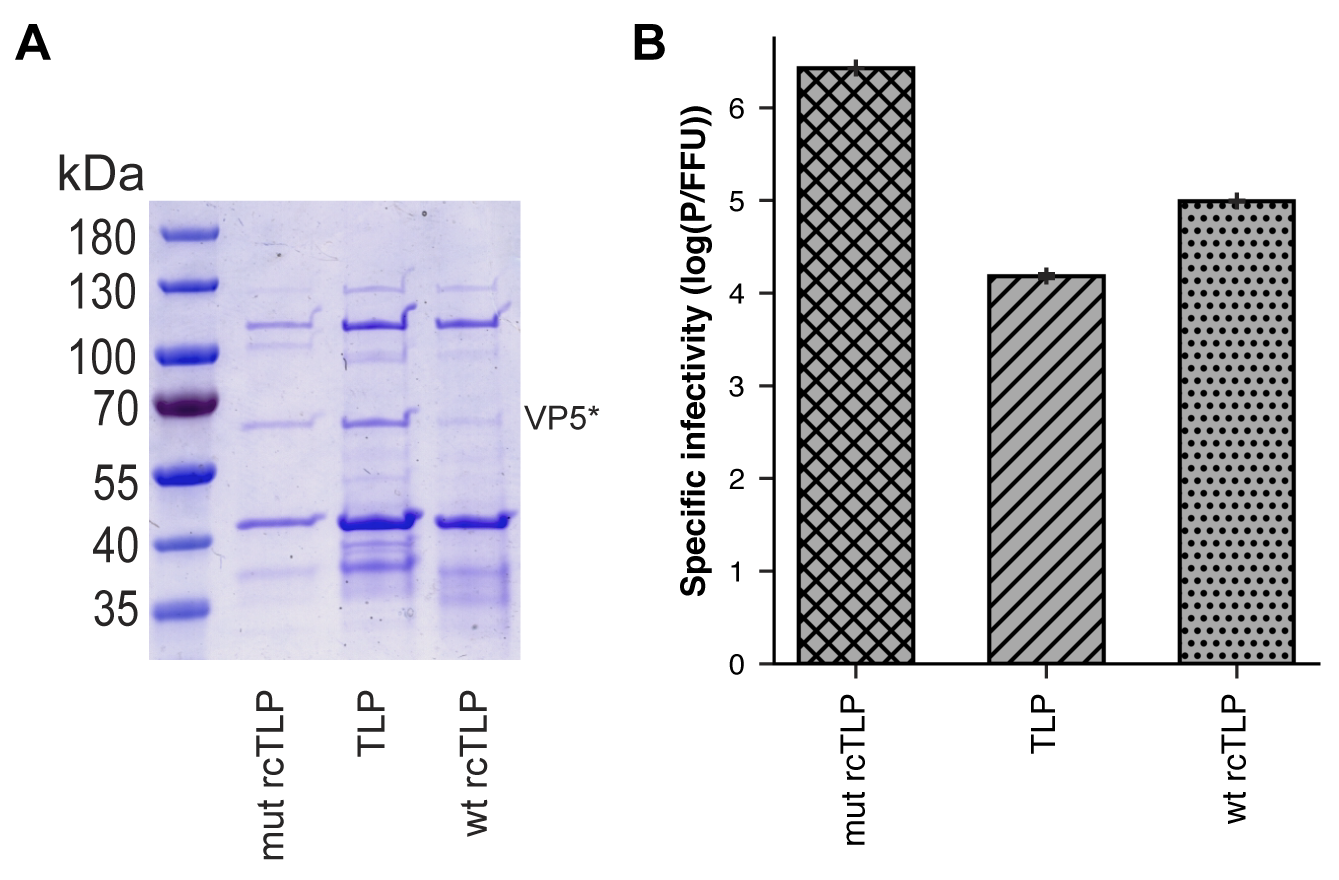

Supplement: S4 Fig — (A) Coomassie-blue stained SDS-PAGE gel of the mut rcTLP (lane 1), TLP (lane 2) and wt rcTLP (lane 3) samples. A quantitative analysis of band intensity, using Fiji [50] and normalizing by the VP6 band intensity, estimated that the wt rcTLPs had only ~25% of theVP5* present in TLPs from cells and that the mut rcTLPs had ~30% more. Wild-type recoating varies considerably from preparation to preparation; the one used here had relatively low occupancy. (B) Particle to focus-forming unit (P/FFU) ratios for mut rcTLP, TLP and wt rcTLP samples. The bar represents the average for 2 measurements and error bars represent the standard deviation. (TIF) [file ppat.1011750.s004.tif]

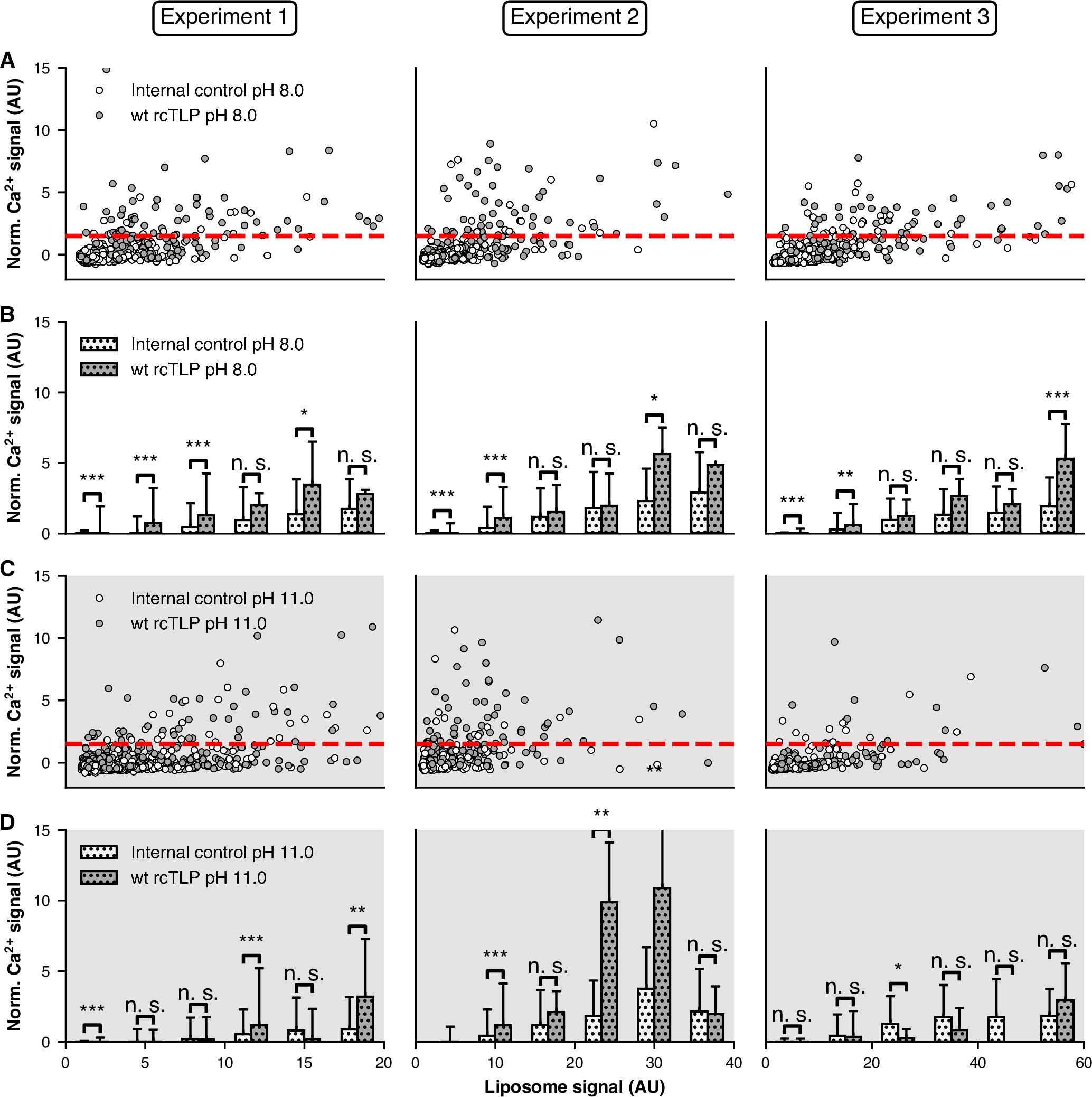

Supplement: S5 Fig — (A) Ca2+ signal intensity (arbitrary units, AU) as a function of liposome signal intensity (AU) for the internal control in the wt rcTLP sample (random selection of liposomes that did not colocalize with virus in the wt rcTLP sample, white dots) and for wt rcTLP (liposomes colocalizing with virus only, grey dots) pre-incubated at pH 8.0, in experiments 1, 2 and 3, respectively. (B) Ca2+ signal intensity (AU) as a function of the binned liposome signal for the internal control in the wt rcTLP sample (dotted white bars) and for wt rcTLP (dotted grey bars) pre-incubated at pH 8.0, in experiments 1, 2 and 3, respectively. Error bars represent the standard deviation calculated from individual measurements within each bin as shown in the scatter plot in panel A. (C) Ca2+ signal intensity (AU) as a function of liposome signal for the internal control in the wt rcTLP sample (random selection of liposomes that did not colocalize with virus in the wt rcTLP sample, white dots) and for wt rcTLP (liposomes colocalizing with virus only, grey dots) pre-incubated at pH 11.0, in experiments 1, 2 and 3, respectively. (D) Ca2+ signal intensity (AU) as a function of the binned liposome signal for the control in the wt rcTLP sample (dotted white bars) and for wt rcTLP (dotted grey bars) pre-incubated at pH 11.0, in experiments 1, 2, and 3, respectively. Error bars represent the standard deviation calculated from individual measurements within each bin as shown in the scatter plot in panel C. Statistical analysis was performed in B and D using a t-test between samples. n.s. = p>0.05; * = p<0.05; ** = p<0.01; *** = p<0.001. (TIF) [file ppat.1011750.s005.tif]

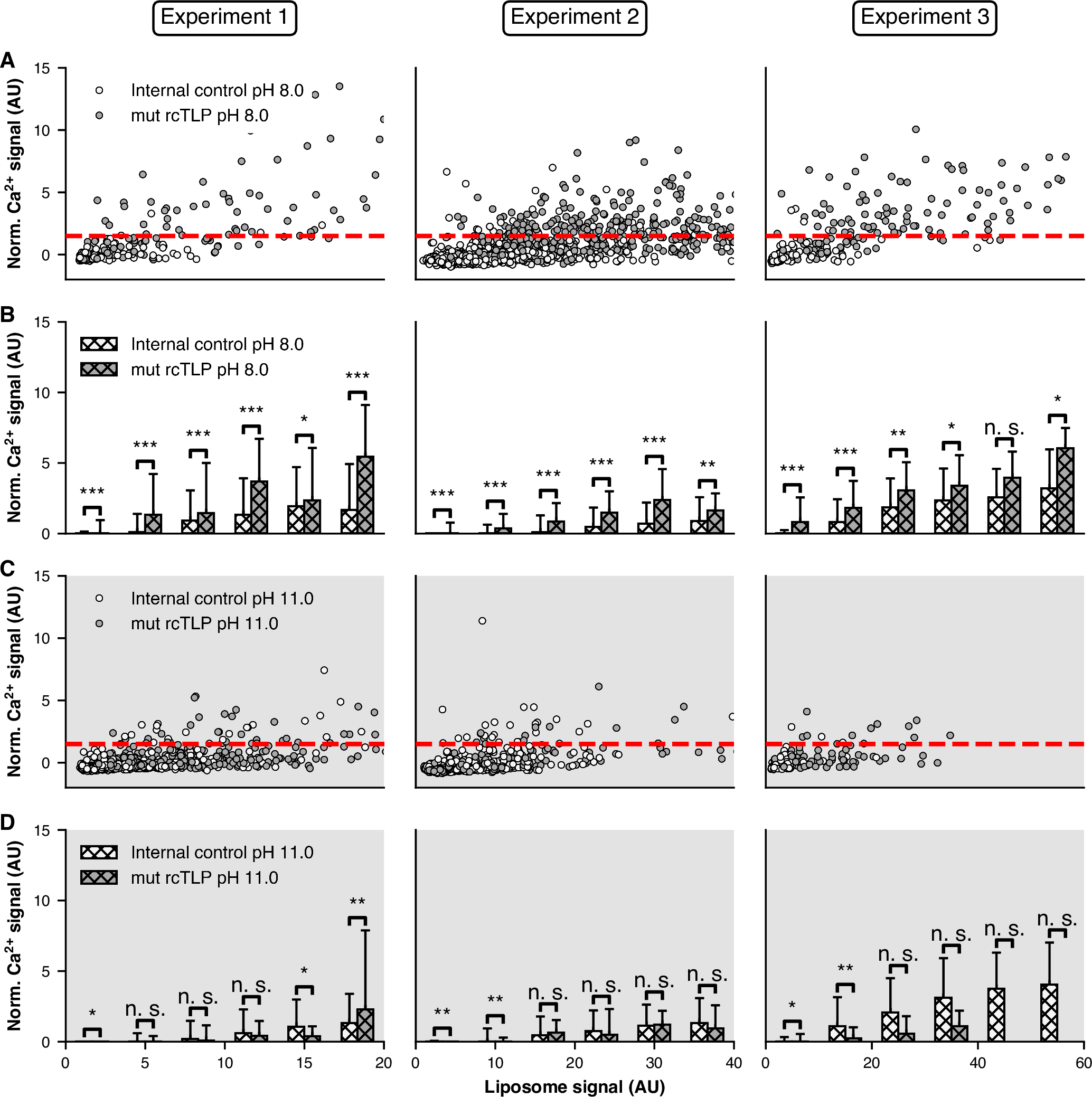

Supplement: S6 Fig — (A) Ca2+ signal intensity (arbitrary units, AU) as a function of liposome signal intensity (AU) for the internal control in the mutant rcTLP sample (random selection of liposomes that did not colocalize with virus in the mutant rcTLP sample: white dots) and for mutant rcTLP (liposomes colocalizing with virus only, grey dots) pre-incubated at pH 8.0, in experiments 1, 2 and 3, respectively. (B) Ca2+ signal intensity as a function of the binned liposome signal for the internal control in the mutant rcTLP sample (cross hatched white bars) and the mutant rcTLP (cross hatched grey bars) pre-incubated at pH 8.0, in experiments 1, 2 and 3, respectively. Error bars represent the standard deviation calculated from individual measurements within each bin as shown in the scatter plot in panel A. (C) Ca2+ signal intensity (AU) as a function of liposome signal for the internal control in the mutant rcTLP sample (random selection of liposomes that did not colocalize with virus in the mutant rcTLP sample: dots in white bar) and for mutant rcTLP (liposomes colocalizing with virus only, dots in gray bar) pre-incubated at pH 11.0, in experiments 1, 2 and 3, respectively. (D) Ca2+ signal intensity as a function of the binned liposome signal for the mutant rcTLP internal control (cross hatched white bars) and the mutant rcTLP sample (liposomes colocalizing with virus only, cross hatched grey bars) pre-incubated at pH 11.0, in experiments 1, 2 and 3, respectively. Error bars represent the standard deviation calculated from individual measurements within each bin as shown in the scatter plot in panel C. Statistical analysis was performed in B and D using a t-test between samples. n.s. = p>0.05; * = p<0.05; ** = p<0.01; *** = p<0.001. (TIF) [file ppat.1011750.s006.tif]

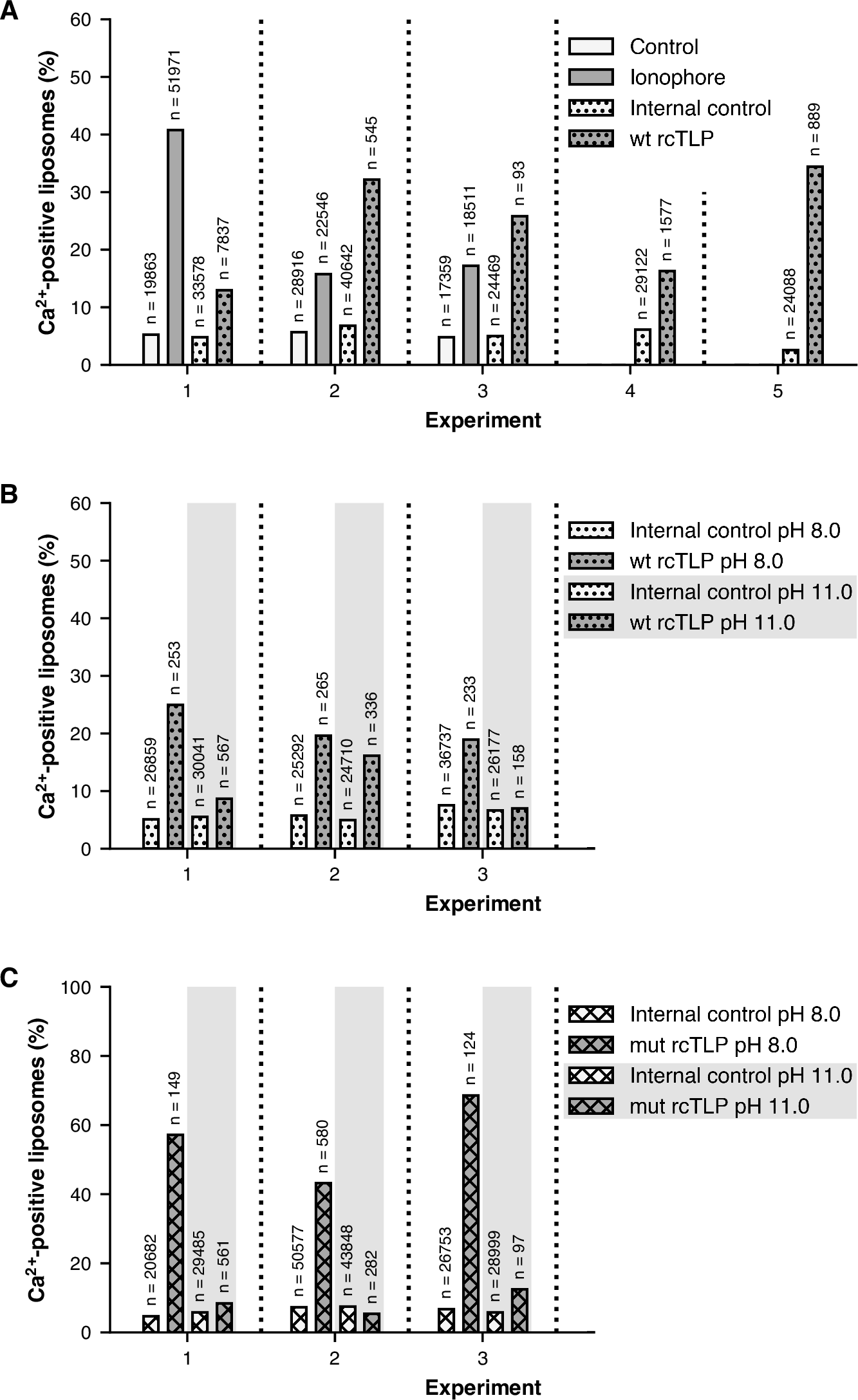

Supplement: S7 Fig — (A) Percent Ca2+-positive liposomes for control liposomes (white bars), ionophore-exposed liposomes (grey bars), internal control in the wt rcTLP sample (liposomes in the wt rcTLP sample that did not colocalize with wt rcTLP: dotted white bars) and wt rcTLP sample (liposomes that colocalized with wt rcTLP: dotted grey bars) from experiments 1, 2, 3, 4 and 5, respectively (S3 Fig). (B) Percent Ca2+-positive liposomes for the internal control in the wt rcTLP sample and wt rcTLP pre-incubated at pH 8.0 (dotted white and dotted grey bars, respectively), and for the internal control in the wt rcTLP sample and wt rcTLP pre-incubated at pH 11.0 (dotted white and dotted grey bars on grey background, respectively) from experiments 1, 2 and 3, respectively (S5 Fig). (C) Ca2+-positive liposomes for the internal control in the mutant rcTLP sample and mutant rcTLP pre-incubated at pH 8.0 (crossed hatched white and crossed hatched grey bars, respectively), and for the internal control in the mutant rcTLP sample and mutant rcTLP pre-incubated at pH 11.0 samples (cross hatched white and cross hatched grey bars on grey background, respectively) from experiments 1, 2 and 3, respectively (S6 Fig). "Ca2+-positive liposomes" designates the percent of toal liposomes scored that had a Ca2+ signal intensity greater than 1.5 standard deviations from the mean in the corresponding control sample (see red dashed lines in S3A, S3C, S5A, S5C, S6A and S6C Figs for the cutoffs). (TIF) [file ppat.1011750.s007.tif]

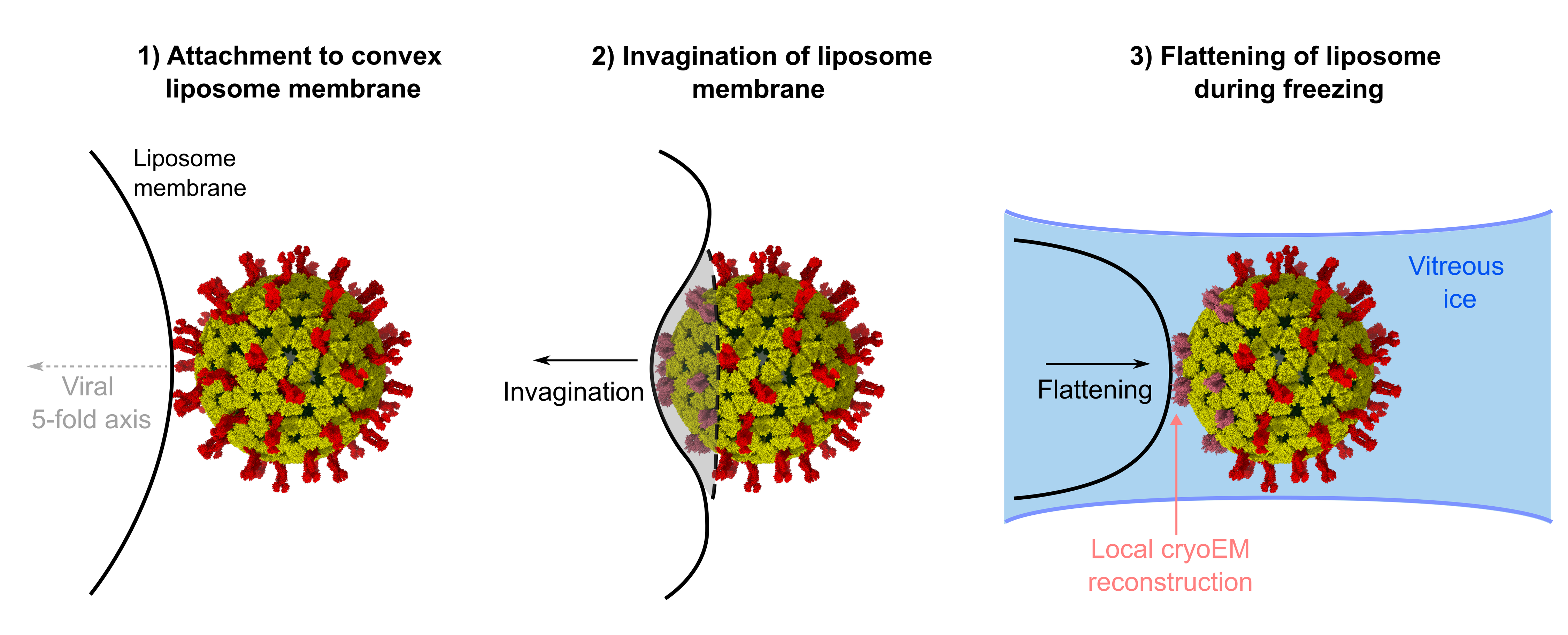

Supplement: S8 Fig — Schematic illustration of the interaction between rotavirus and a liposome during specimen preparation for cryo-EM analysis. (TIF) [file ppat.1011750.s008.tif]

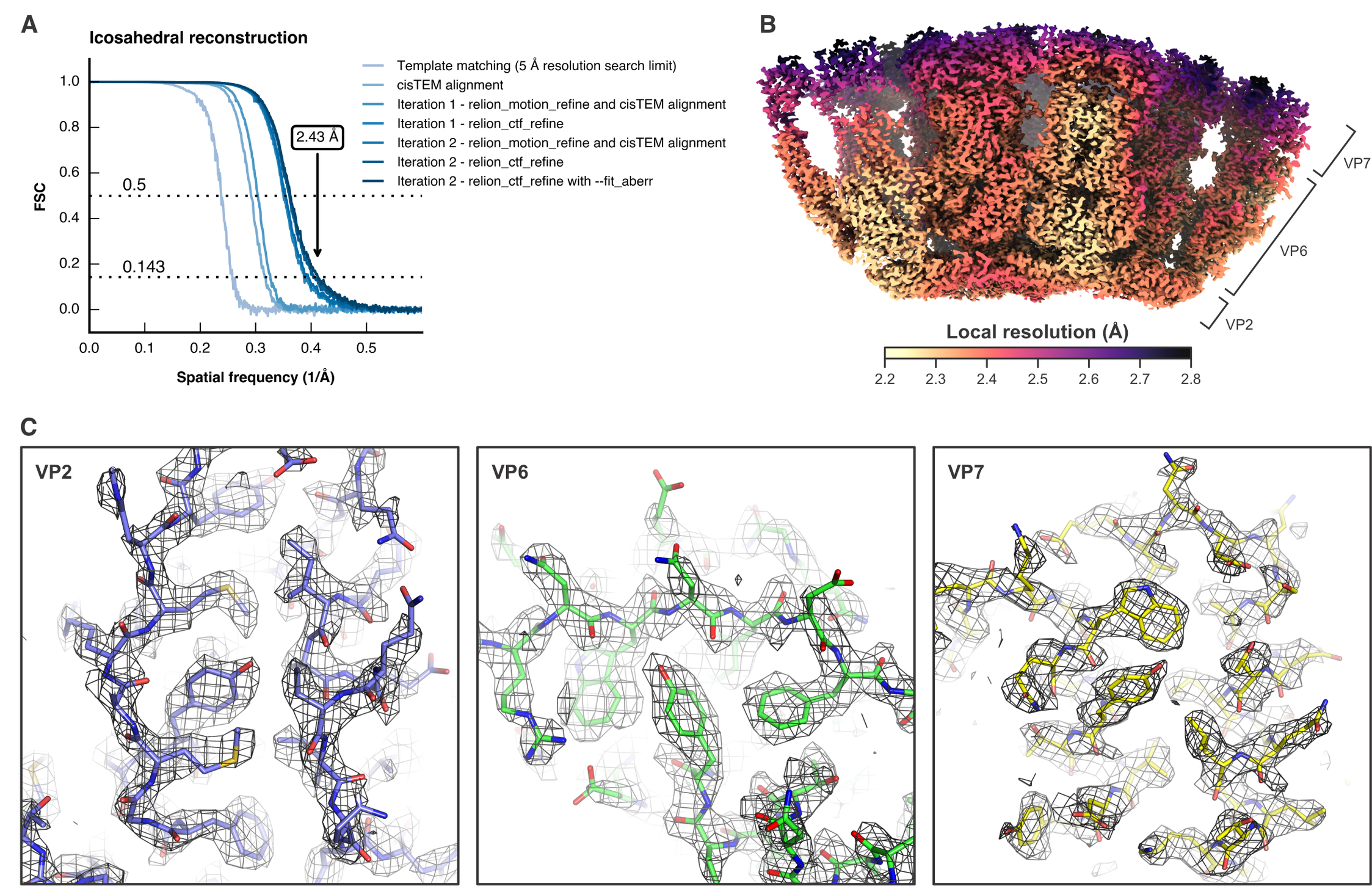

Supplement: S9 Fig — (A) Fourier shell correlations (FSCs) between half maps calculated from icosahedral reconstructions after applying a spherical shell mask encompassing the three protein layers of the TLP (VP2, VP6, and VP7). Curves are shown for the data processing steps as described in Methods. The final nominal resolution was 2.43 Å. (B) Local resolution analysis of the icosahedral reconstruction. A segment of the “triple-layer” particle is shown. (C) Density maps of VP2, VP6 and VP7 regions. (TIF) [file ppat.1011750.s009.tif]

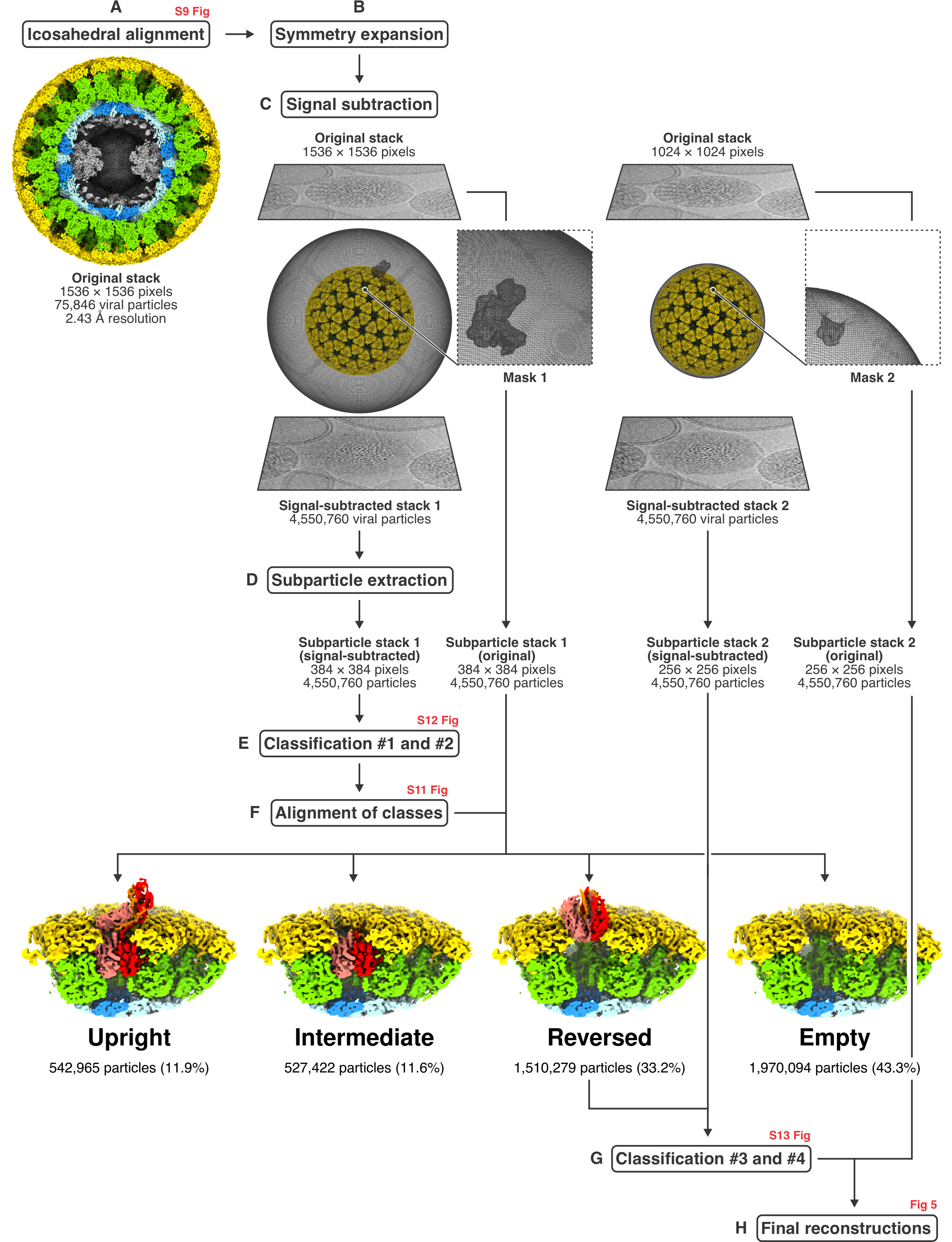

Supplement: S10 Fig — (A) Alignment of full viral particles with icosahedral symmetry imposed. A map of the final icosahedral reconstruction is shown where the particle is partially cut. VP1 (RNA-dependent RNA polymerase) and RNAs are colored gray; VP2, cyan and blue; VP6, green; VP7, yellow. See also S9 Fig. (B) Icosahedral symmetry expansion (60-fold) based on the full particle alignment. (C) Signal subtraction. The masks used to the define the subtraction volume, excluding the region of interest at a single protomer position, are shown. Signal-subtracted stack 1 was used to prepare subparticles for classifications #1 and #2 (spike conformation and occupancy). Signal-subtracted stack 2 was used to prepare subparticles for classifications #3 and #4 (liposome interaction of reversed spikes). (D) Subparticle extraction from the signal-subtracted and original particles stacks. (E) Classifications #1 and #2, see S12 Fig. (F) Alignment of classes to correct for observed shifts in reconstructions of subparticle classes caused primarily by anisotropic magnification distortion, see S11 Fig. Subparticle reconstructions are shown for the three observed spike conformations (upright, intermediate, and reversed) and empty positions. Density maps were low pass filtered at 5 Å resolution and partially cut. VP2, cyan and blue; VP6, green; VP7, yellow; VP5*, red, orange, and salmon. (G) Classifications #3 and #4, see S13 Fig. (H) Final reconstructions of liposome-bound classes calculated from subparticle stack 2, which was obtained from the original (non-signal-subtracted) images. (TIF) [file ppat.1011750.s010.tif]

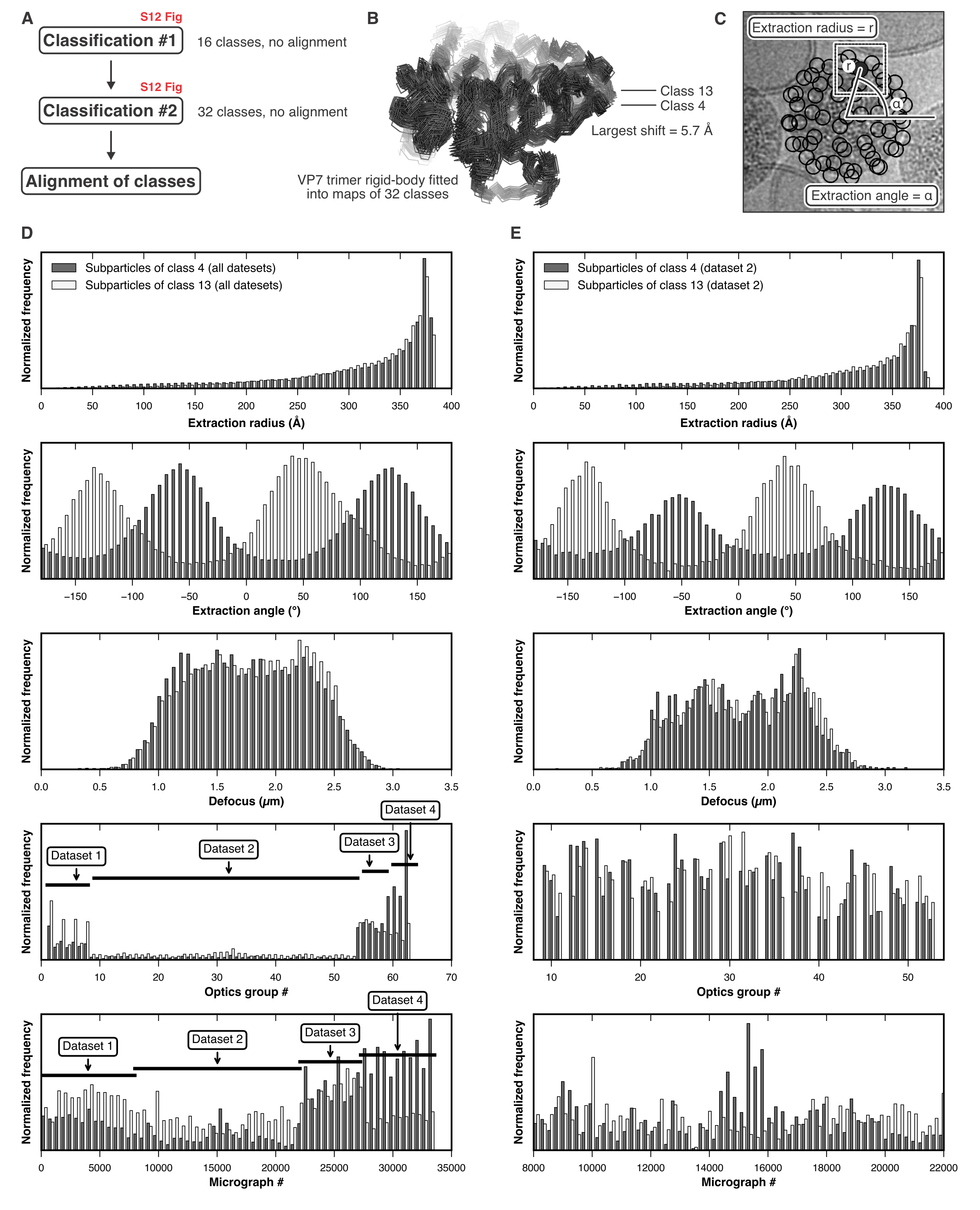

Supplement: S11 Fig — (A) Subparticles were classified without alignment. Relative shifts between classes were determined by rigid-body fitting a V7 trimer model and then aligning the classes by updating the subparticle alignment parameters based on the fitted models. (B) Ribbon representation of the VP7 trimer models after fitting it into the densities of the 32 classes. The largest shift we observed was 5.7 Å between classes 4 and 13. (C) Each subparticle is associated with an extraction radius and angle with respect to the center of the full virus particle projection in the original micrograph. (D) Histograms of metadata values (extraction radius, extraction angle, defocus, optics group number, micrograph number) for all subparticles from the two classes 4 and 13, which showed the largest relative shift in their reconstructions. A strong bias in the extraction angle suggests that the observed shifts are predominately caused by anisotropic magnification distortion, which was not accounted for during subparticle extraction. (D) Histograms of metadata values (extraction radius, extraction angle, defocus, optics group number, micrograph number) for dataset 2 subparticles from the two classes 4 and 13. (TIF) [file ppat.1011750.s011.tif]

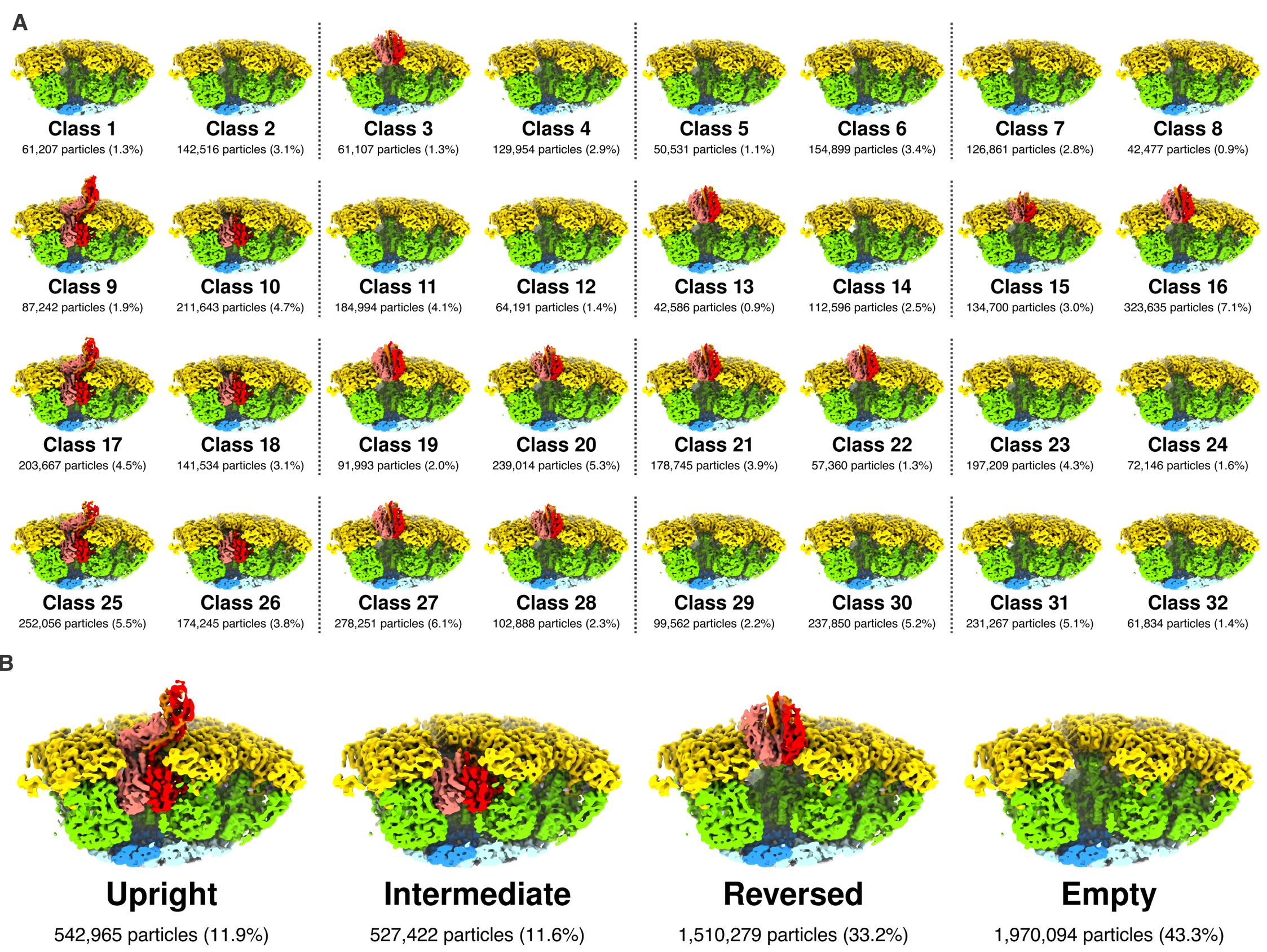

Supplement: S12 Fig — For display, density maps were calculated from subparticle stack 1, which was obtained from the original (non-signal-subtracted) images, low pass filtered at 5 Å resolution and partially cut. VP2, cyan and blue; VP6, green; VP7, yellow; VP5*, red, orange, and salmon. (A) Classification #1 partitioned the particles into 16 classes, each of which was further subclassified into two classes in classification #2 (indicated by dashed lines). (B) Reconstructions of three observed spike conformations (upright, intermediate, and reversed) and empty positions after merging corresponding classes (S2 Table) and correcting of observed subparticle shifts (S11 Fig). (TIF) [file ppat.1011750.s012.tif]

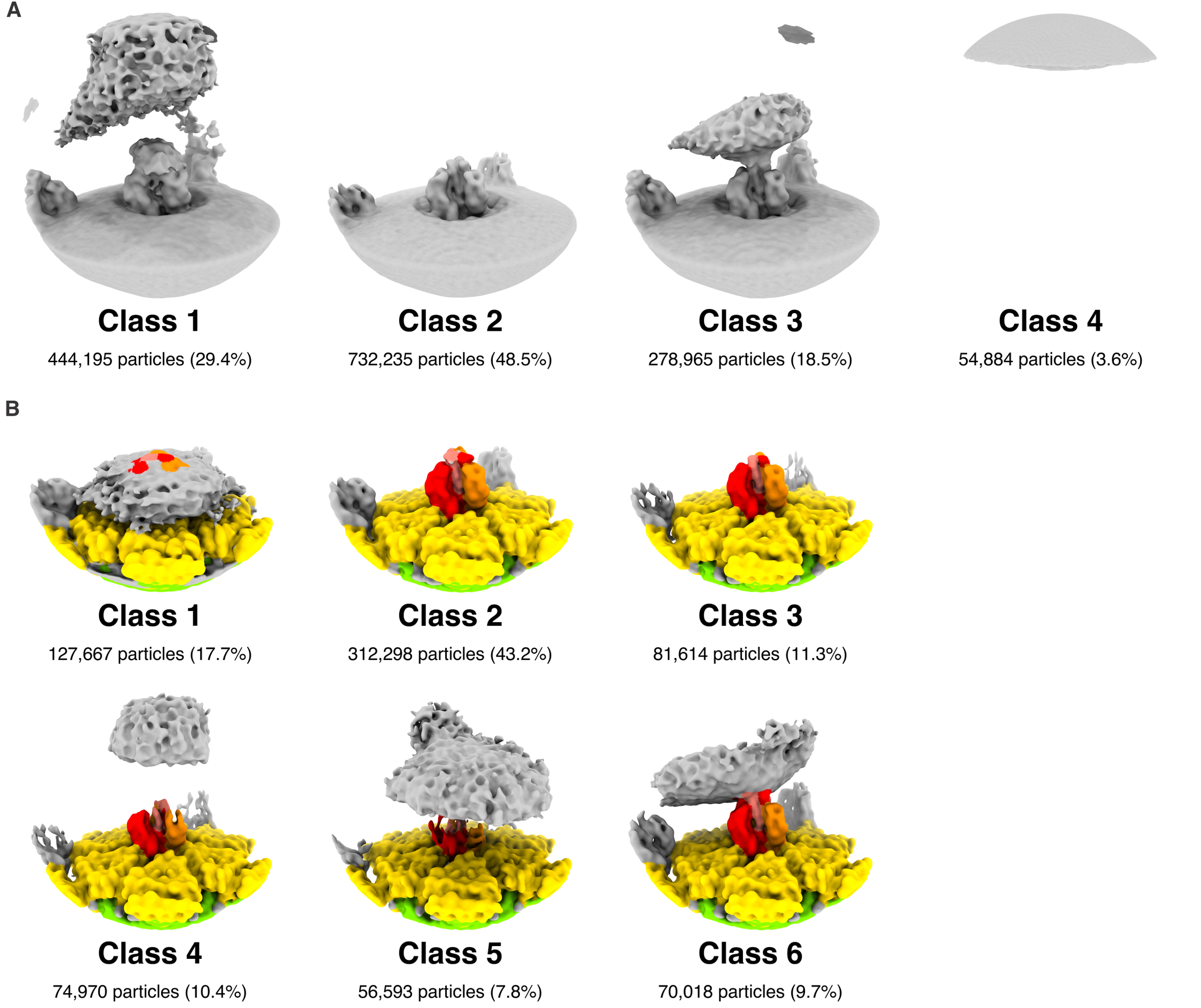

Supplement: S13 Fig — Classification of liposome-bound spike positions from subparticles with reversed spike conformations. (A) In classification #3, we initially requested four classes. Density maps were calculated from signal-subtracted subparticle stack 2, low pass filtered at 8 Å resolution and displayed in gray and at very low contour level to visualize membrane density. (B) In classification #4, we further subclassified classes 1 and 3 from classification #3. Density maps of the six classes were calculated from subparticle stack 2, which was obtained from the original (non-signal-subtracted) images, and low pass filtered at 8 Å. VP6, green; VP7, yellow; VP5*, red, orange, and salmon; membrane, gray. (TIF) [file ppat.1011750.s013.tif]

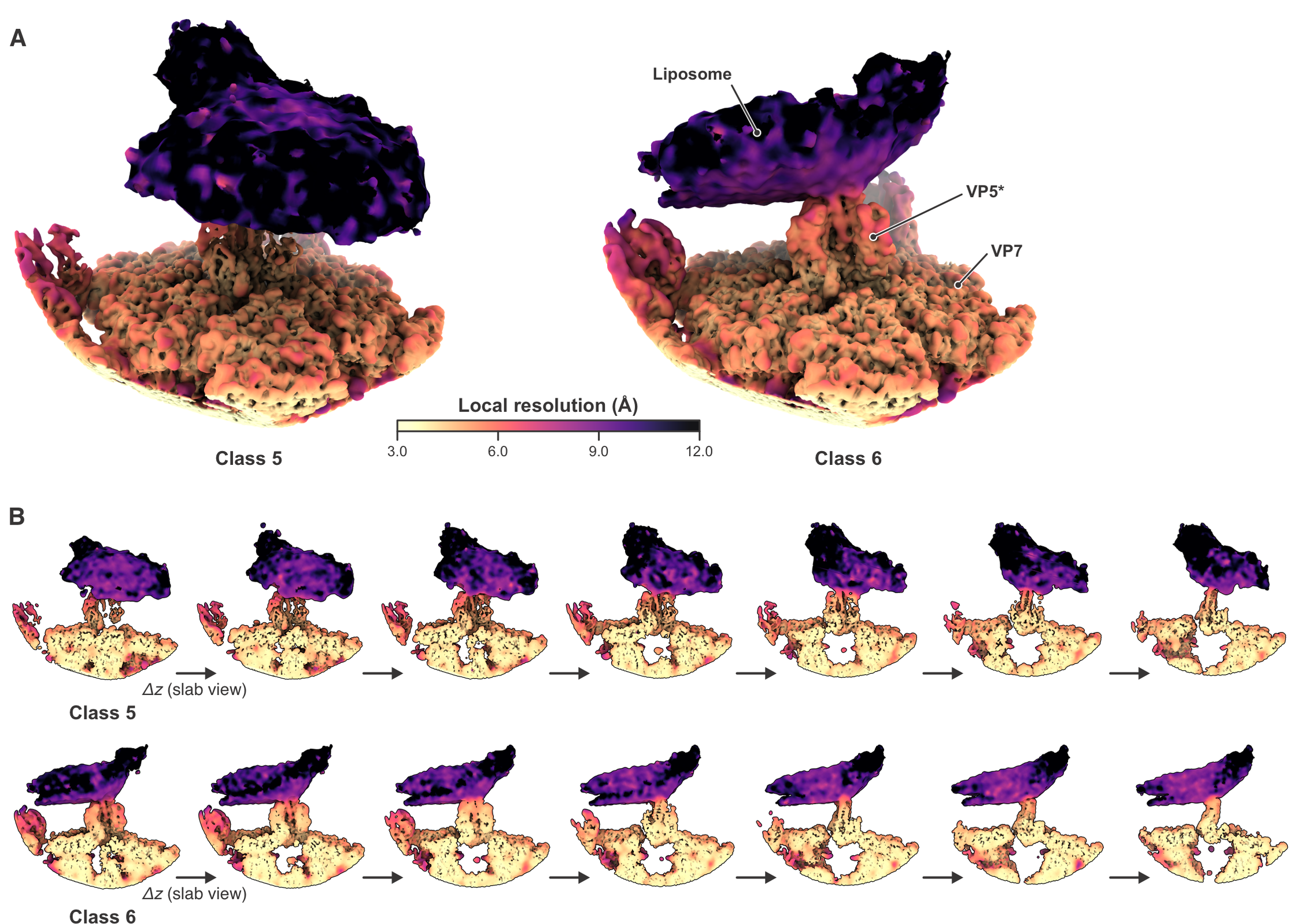

Supplement: S14 Fig — (A) Full view of the class 5 and class 6 reconstructions colored according to local resolution. (B) Corresponding slab views. (TIF) [file ppat.1011750.s014.tif]

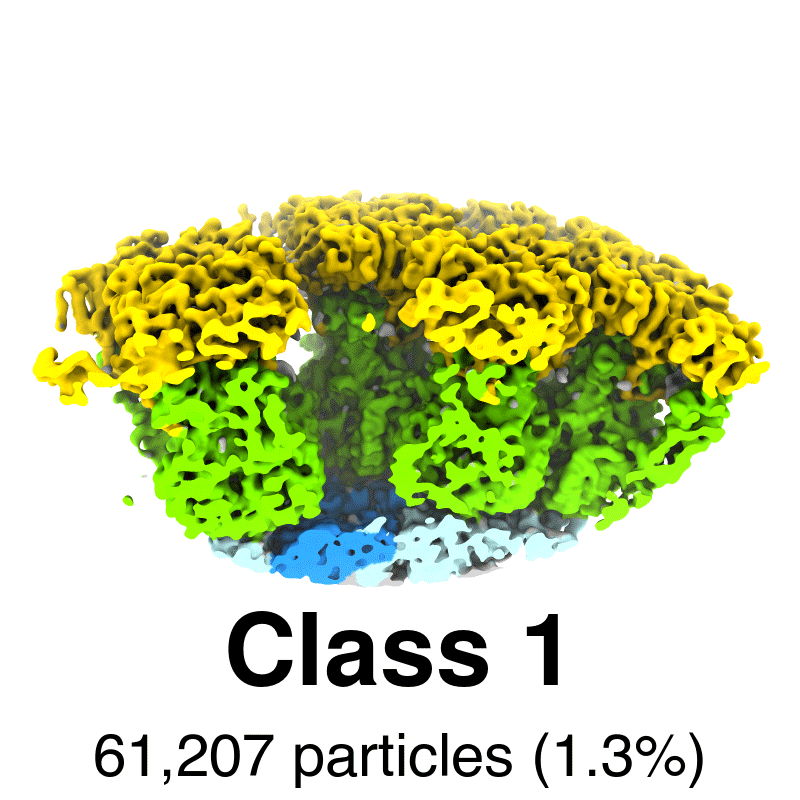

Supplement: S1 Movie — (GIF) [file ppat.1011750.s019.gif]
