# Supplementary material for: The rotavirus VP5*/VP8* conformational transition permeabilizes membranes to Ca2+
Source: PLoS Pathog. 2024 Apr 4;20(4):e1011750. doi: 10.1371/journal.ppat.1011750 (PMC11020617; doi:10.1371/journal.ppat.1011750)
Supplement: S1 Table — (PDF) [file ppat.1011750.s015.pdf]

S1 Table. Cryo-EM data collection and statistics

	Dataset 1<br>RRV liposomes	Dataset 2<br>RRV liposomes	Dataset 3<br>RRV liposomes	Dataset 4<br>RRV liposomes
**Data collection**				
Electron microscope	Titan Krios	Titan Krios	Titan Krios	Titan Krios
Camera	K3 Summit	K3 Summit	K3 Summit	K3 Summit
Magnification	60,606	60,606	60,606	60,606
Voltage (kV)	300	300	300	300
Number of movies	7,985	14,088	5,170	6,320
Defocus range (μm) *	1.0–2.5	1.0–2.5	1.0–2.5	1.0–2.5
Pixel size (Å)	0.825	0.825	0.825	0.825
**Icosahedral reconstruction**				
Number of images	75,846			
Box size (pixels)	1536			
Pixel size (Å)	0.825			
Symmetry imposed	I (setting I2)			
Map resolution (Å) †	2.43			
	**Subparticle stack 1**	**Subparticle stack 2**	**Final class 5**<br>EMD-42343<br>PDB-ID 8UK2	**Final class 6**<br>EMD-42344<br>PDB-ID 8UK3
**Local reconstruction**				
Number of images	4,550,760	4,550,760	56,593	70,018
Box size (pixels)	385	256	256	256
Pixel size (Å)	0.825	1.2375	1.2375	1.2375
Symmetry imposed	C_1	C_1	C_1	C_1
Map resolution (Å)	2.91, 2.73 ‡	3.33, 3.14 §	N.A.	N.A.

* Approximate range of underfocus.
† Resolution where Fourier shell correlation (FSC) between half-maps drops below 0.143 after applying a spherical shell mask (inner radius = 222 Å, outer radius = 403 Å).
‡ Resolution after applying a mask encompassing one VP7 trimer and the volume corresponding to VP4 in upright and reversed conformation for reconstructions from the original (non-signal-subtracted) subparticle stack 1 before and after alignment of classes 1–32, respectively.
§ Resolution after applying a mask encompassing volume corresponding to VP4 in upright and reversed conformation for reconstructions from the original (non-signal-subtracted) subparticle stack 2 before and after alignment of classes 1–32, respectively.
