# Supplementary material for: The rotavirus VP5*/VP8* conformational transition permeabilizes membranes to Ca2+
Source: PLoS Pathog. 2024 Apr 4;20(4):e1011750. doi: 10.1371/journal.ppat.1011750 (PMC11020617; doi:10.1371/journal.ppat.1011750)
Supplement: S2 Table — (PDF) [file ppat.1011750.s016.pdf]

S2 Table. Classification #1 and #2 of VP5*/VP8* spike positions

Classification #1 *			Classification #2 †				
1	203,723	4.5%	1	1	61207	1.3%	Empty (partially occupied)
			2	2	142516	3.1%	Empty
2	191,061	4.2%	1	3	61107	1.3%	Reversed
			2	4	129954	2.9%	Empty
3	205,430	4.5%	1	5	50531	1.1%	Empty
			2	6	154899	3.4%	Empty
4	169,338	3.7%	1	7	126861	2.8%	Empty
			2	8	42477	0.9%	Empty
5	298,885	6.6%	1	9	87242	1.9%	Upright
			2	10	211643	4.7%	Intermediate (β-barrel domains disordered)
6	249,185	5.5%	1	11	184994	4.1%	Empty
			2	12	64191	1.4%	Empty
7	155,182	3.4%	1	13	42586	0.9%	Reversed
			2	14	112596	2.5%	Empty
8	458,335	10.1%	1	15	134700	3.0%	Reversed
			2	16	323635	7.1%	Reversed
9	345,201	7.6%	1	17	203667	4.5%	Upright
			2	18	141534	3.1%	Upright (β-barrel domains disordered)
10	331,007	7.3%	1	19	91993	2.0%	Reversed
			2	20	239014	5.3%	Reversed
11	236,105	5.2%	1	21	178745	3.9%	Reversed
			2	22	57360	1.3%	Reversed
12	269,355	5.9%	1	23	197209	4.3%	Empty
			2	24	72146	1.6%	Empty (partially occupied)
13	426,301	9.4%	1	25	252056	5.5%	Upright
			2	26	174245	3.8%	Intermediate (β-barrel domains disordered)
14	381,139	8.4%	1	27	278251	6.1%	Reversed
			2	28	102888	2.3%	Reversed
15	337,412	7.4%	1	29	99562	2.2%	Empty
			2	30	237850	5.2%	Empty
16	293,101	6.4%	1	31	231267	5.1%	Empty
			2	32	61834	1.4%	Empty
					**542,965**	**11.9%**	**Upright**
					**527,422**	**11.6%**	**Intermediate**
					**1,510,279**	**33.2%**	**Reversed**
					**1,970,094**	**43.3%**	**Empty**
					**4,550,760**	**100%**	**Total**

∗ Columns are class number of classification #1, number of particles, percentage of particles.
† Columns are class number of classification #2, final class number, number of particles, percentage of particles, occupancy state and conformation.
