# Supplementary material for: The rotavirus VP5*/VP8* conformational transition permeabilizes membranes to Ca2+
Source: PLoS Pathog. 2024 Apr 4;20(4):e1011750. doi: 10.1371/journal.ppat.1011750 (PMC11020617; doi:10.1371/journal.ppat.1011750)
Supplement: S3 Table — (PDF) [file ppat.1011750.s017.pdf]

S3 Table. Classification #3 and #4 of reversed VP5*/VP8* spikes

Classification #3 *			
1	444,195	29.4%	Liposome density
2	732,235	48.5%	No liposome density
3	278,965	18.5%	Liposome density
4	54,884	3.6%	Junk
	**1,510,279**	**100%**	**Total**

Classification #4 *			
1	127,667	17.7%	Liposome density
2	312,298	43.2%	No liposome density
3	81,614	11.3%	No liposome density
4	74,970	10.4%	Liposome density
5	56,593	7.8%	Liposome density
6	70,018	9.7%	Liposome density
	**723,160**	**100%**	**Total**

∗ Columns are class number, number of particles, percentage of particles, assignment.
