# Supplementary material for: The rotavirus VP5*/VP8* conformational transition permeabilizes membranes to Ca2+
Source: PLoS Pathog. 2024 Apr 4;20(4):e1011750. doi: 10.1371/journal.ppat.1011750 (PMC11020617; doi:10.1371/journal.ppat.1011750)
Supplement: S4 Table — (PDF) [file ppat.1011750.s018.pdf]

S4 Table. VP5*/VP8* spike conformations in liposome-bound versus liposome-unbound rcTLPs

rcTLPs selection	#	Upright (%)	Intermediate (%)	Reversed (%)	Empty (%)
All rcTLPs	75846	11.9	11.6	33.2	43.3
Random selection of rcTLPs	35435	11.9	11.6	33.2	43.3
Liposome-unbound rcTLPs (5) ∗	34352	11.2	10.7	30.3	47.8
Liposome-unbound rcTLPs (6) †	35528	10.9	10.1	29.1	49.9
**Average liposome-unbound ‡**		**11.1 (21.6)**	**10.4 (20.3)**	**29.7 (58.1)**	**48.9**
Liposome-bound rcTLPs (5) ∗	41494	12.8	12.7	36.7	37.9
Liposome-bound rcTLPs (6) †	40318	13.2	13.2	37.8	35.8
**Average liposome-bound ‡**		**13.0 (20.6)**	**13.0 (20.5)**	**37.3 (58.9)**	**36.9**

∗ Based on class #5 (Figure S13B). Liposome-unbound rcTLPs did not contribute any subparticles to class #5, liposome-bound rcTLPs contributed at least one subparticle to class #5.

† Based on class #6 (Figure S13B). Liposome-unbound rcTLPs did not contribute any subparticles to class #6, liposome-bound rcTLPs contributed at least one subparticle to class #6.

‡ Values in parenthesis are the percentages with respect to occupied VP5*/VP8* positions only.
